# Health outcomes and service use patterns associated with co‐located outpatient mental health care and alcohol and other drug specialist treatment: A systematic review

**DOI:** 10.1111/dar.13651

**Published:** 2023-04-04

**Authors:** Clare Glover‐Wright, Kym Coupe, Alexander Charles Campbell, Claire Keen, Patrick Lawrence, Stuart A. Kinner, Jesse T. Young

**Affiliations:** ^1^ Centre for Health Equity, Melbourne School of Population and Global Health The University of Melbourne Melbourne Australia; ^2^ First Step Melbourne Australia; ^3^ Centre for Adolescent Health, Murdoch Children's Research Institute Melbourne Australia; ^4^ School of Population Health Curtin University Perth Australia; ^5^ Griffith Criminology Institute Griffith University Brisbane Australia; ^6^ School of Population and Global Health The University of Western Australia Perth Australia; ^7^ National Drug Research Institute Curtin University Perth Australia

**Keywords:** alcohol and other drug treatment, community mental health services, diagnosis, dual (psychiatry), mental disorders, systematic review

## Abstract

**Issues:**

Despite long‐standing recommendations to integrate mental health care and alcohol and other drug (AOD) treatment, no prior study has synthesised evidence on the impact of physically co‐locating these specialist services on health outcomes.

**Approach:**

We searched Medline, PsycINFO, Embase, Web of Science and CINAHL for studies examining health outcomes associated with co‐located outpatient mental health care and AOD specialist treatment for adults with a dual diagnosis of substance use disorder and mental illness. Due to diversity in study designs, patient populations and outcome measures among the included studies, we conducted a narrative synthesis. Risk of bias was assessed using the MASTER scale.

**Key Findings:**

Twenty‐eight studies met our inclusion criteria. We found provisional evidence that integrated care that includes co‐located mental health care and AOD specialist treatment is associated with reductions in substance use and related harms and mental health symptom severity, improved quality of life, decreased emergency department presentations/hospital admissions and reduced health system expenditure. Many studies had a relatively high risk of bias and it was not possible to disaggregate the independent effect of physical co‐location from other common aspects of integrated care models such as care coordination and the integration of service processes.

**Implications:**

There are few high‐quality, peer‐reviewed studies establishing the impact of co‐located mental health care and AOD specialist treatment on health outcomes. Further research is required to inform policy, guide implementation and optimise practice.

**Conclusion:**

Integrated care that includes the co‐location of mental health care and AOD specialist treatment may yield health and economic benefits.

**Key Points**

- There is consistent evidence that integrated care that includes co‐located mental health care and alcohol and other drug (AOD) specialist treatment was positively correlated with improved patterns of treatment engagement and remission or abstinence from substance use.
- There is a limited body of evidence that integrated care that includes co‐located mental health and AOD specialist treatment is associated with reduced mental health symptom severity and decreased rates of emergency department presentations and hospitalisations.
- Most co‐located service models differed from the comparison models of care beyond co‐location, including differing levels of care coordination, integration of service provider processes and clinical service team composition.
- Evidence reported in the included studies did not allow us to isolate the independent effect of physical co‐location, therefore our findings are most accurately interpreted as outcomes related to integrated care models that include co‐location of mental health and AOD specialist treatment.
- Evidence in the included studies was heterogeneous, relatively limited in scope, based almost exclusively on samples from the United States, and many studies had a relatively high risk of bias.

## 1 INTRODUCTION

Substance use disorder and mental illness frequently co‐occur [1, 2], can arise simultaneously or sequentially [3, 4] and are strongly associated, whereby the presence of one of these conditions increases the likelihood of developing the other [5, 6]. The co‐occurrence of substance use disorder and mental illness, often referred to as dual diagnosis, is estimated to affect approximately 2% of the general population [7, 8].

People with dual diagnosis experience rates of premature mortality between 5.0 and 8.5 times higher than that in the general population and double that observed among people with mental illness alone [9, 10]. Dual diagnosis has been associated with increased self‐harm [11], suicidality [12], interpersonal violence perpetration [13], non‐fatal overdose and other non‐fatal injury [14, 15], higher rates of co‐occurring chronic physical conditions [16, 17], disability [18] and reduced quality of life [19], which combined with increased unemployment [20], homelessness [21] and criminal justice system involvement [22, 23], present challenges for effective management and treatment.

People with dual diagnosis frequently report increased structural, financial, psychosocial and model of care‐related barriers to accessing specialist care [24, 25]. Continuity of care, defined as ‘the degree to which a series of healthcare events is experienced as coherent, connected and consistent’ ([26]; p. 1220), is associated with better quality of life and social functioning, reduced psychiatric symptom severity, abstinence from substance use, improved physical and mental health, decreased risk of hospitalisation, lower total health‐care costs and decreased premature mortality [27, 28, 29, 30, 31, 32]. However, in many high‐income countries (e.g., Canada, Australia and the United States), the historical separation between mental health and alcohol and other drug (AOD) service systems [33, 34, 35] is a prominent barrier to continuity of care for those with dual diagnosis. Due to these barriers, people with dual diagnosis report increased unmet need for mental health and AOD treatment [24, 36], and experience more complex symptomatology, increased disorder chronicity, lower rates of abstinence from substance use, higher rates of treatment attrition, and more adverse psychological and pharmacotherapy‐related events compared to those with mental illness or substance use disorder alone [37, 38, 39]. To address these barriers and improve outcomes for people with dual diagnosis, integrated models of mental health care and AOD specialist treatment have been developed and implemented [40] and most guidelines for the management of dual diagnosis recommend an integrated approach to mental health and AOD specialist treatment [41].

Common elements of integrated care models include: (i) care coordination, whereby care is co‐ordinated between two or more services in more than one location; (ii) the integration of service provider processes, whereby two or more services adapt and share administrative, information and/or financing systems; and (iii) the physical co‐location of two or more services and/or practitioners from multiple disciplines [42]. To optimise the efficacy of these integrated models of care, it is important to understand the unique impact of each element on the outcomes these models intend to achieve.

The co‐location of services inherently removes some of the administrative, organisational and structural barriers to providing integrated, cohesive care for people with dual diagnosis [42, 43]. However, evidence regarding the health outcomes associated with co‐located mental health care and AOD specialist treatment has yet to be systematically synthesised. Furthermore, neither the impact of the co‐location of mental health care and AOD specialist treatment on service use and health‐care expenditure, nor the cost‐effectiveness of these models, is well‐understood. Therefore, by conducting a systematic review of the peer‐reviewed literature, we aimed to examine the: (i) treatment engagement patterns; (ii) substance use patterns and harms; (iii) health outcomes; (iv) service use patterns; and (v) health economic outcomes associated with the co‐location of outpatient mental health care and AOD specialist treatment provided to adults (≥18‐years‐old) with a dual diagnosis of substance use disorder and mental illness.

## 2 METHODS

This systematic review was registered with the International Prospective Register of Systematic Reviews (PROSPERO—ref# CRD42020213358) and is reported in accordance with the Preferred Reporting Items for Systematic Reviews and Meta‐Analysis (PRISMA) guidelines [44].

### 2.1 Search strategy

We used the patient, intervention, comparison, outcome framework to define key search terms relevant to the aims of our study (Table S1, Supporting Information).

Using terms related to dual diagnosis, substance use disorder, mental disorder, co‐location and treatment (Tables S2–S6), we systematically searched Medline, PsycINFO, Embase, Cumulative Index of Nursing and Allied Health Literature (CINAHL), and Web of Science databases for peer‐reviewed studies reported in English. We searched each database from inception until the day of conducting our final search on 24 August 2020. We updated our search on 18 October 2021 prior to article submission. The reference and citation lists of all included studies were checked to ascertain relevant studies not identified by our systematic search strategy.

### 2.2 Study selection

Our inclusion and exclusion criteria are displayed in Table S7. We did not impose restrictions on geographic area, or the timing between exposure (i.e., the time from which a patient is engaged in co‐located treatment) and the occurrence of our outcomes of interest.

Studies identified in the systematic search were imported into EndNote X8 then transferred to Covidence where duplicates were removed. Title and abstracts were independently screened by two research team members. Conflicts were resolved by consensus with a third research team member. Inter‐rater reliability was estimated using Cohen's kappa statistic [45]. The full‐text of all studies included after title and abstract screening was independently reviewed by two research team members. Conflicts were resolved by consensus with a third research team member. If the full‐text could not be sourced, we made two attempts to contact the author(s) via email to request a copy. If we did not receive a response, the study was excluded.

### 2.3 Data extraction

We used a pre‐specified, structured data extraction form to extract information from eligible articles. Where available, we extracted the year(s) of study, country it was conducted in, study design, sample size, age and sex of participants, population clinical characteristics and length of follow‐up. We summarised the included articles using the following service delivery model and outcome domains: (i) models of service co‐location; (ii) comparison service model (if relevant); (iii) treatment engagement patterns; (iv) substance use patterns and harms; (v) health outcomes; (vi) health service use patterns and barriers to accessing health services; and (vii) health economic outcomes. If different studies reported data on the same outcome, the study with the longest follow‐up duration was included for that outcome.

Aligned with these outcome domains, and informed by recommendations of the Economic and Social Research Council Methods Program [46], we conducted a narrative synthesis of the included studies.

### 2.4 Quality assessment

We assessed study quality using the Methodological Standard for Epidemiological Research (MASTER) scale [47]. The MASTER Scale assesses the methodological quality of studies across 40 bias safeguards within seven standards: equal recruitment, equal ascertainment, equal implementation, equal retention, sufficient analysis, equal prognosis and temporal precedence [47]. Each bias safeguard is ranked as either a 0 or 1; 0 if the safeguard is inapplicable or absent and 1 if the safeguard is both applicable and present.

We calculated the total proportion of bias safeguards present for each study. Consistent with the MASTER scale, and to permit comparison of the relative quality of the included studies, we calculated the relative risk of bias score for each included study (i.e., relative to the study with the highest number of bias safeguards present) [47].

### 2.5 Sensitivity analysis

We conducted a sensitivity analysis whereby we restricted our analyses to only those studies with a low risk of bias.

## 3 RESULTS

**FIGURE 1:**
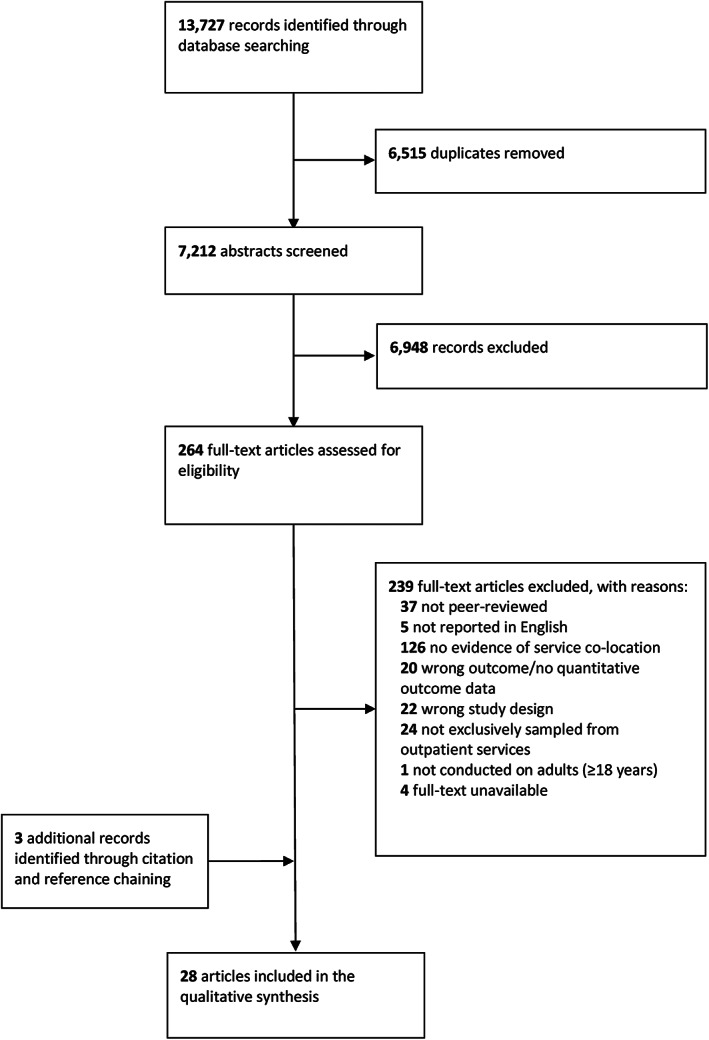
PRISMA flow diagram of article selection.

The results from our systematic search and study selection process are summarised in Figure 1. Our database search identified 13,727 records. After removing 6515 duplicates, 7212 unique articles underwent title and abstract screening. We excluded 6948 articles and the remaining 263 articles were subjected to full‐text screening. Agreement for title and abstract screening between reviewers was moderate (Kappa = 0.41) [45]. We excluded 238 of the 263 studies in full‐text review. The remaining 25 studies were included. We identified three additional studies through reference and citation chaining, yielding a total of 28 studies included in our narrative synthesis (Figure 1).

### 3.1 Characteristics of included studies

The studies included in this review are summarised in Table 1. Twenty‐seven of 28 studies were conducted in the United States [48, 49, 50, 51, 52, 53, 54, 55, 56, 57, 58, 59, 60, 61, 62, 63, 64, 65, 66, 67, 68, 69, 70, 71, 72, 73, 74]. One study was conducted in Norway [75]. Ten of the 28 studies employed a randomised controlled trial design [48, 52, 53, 56, 57, 59, 60, 64, 65, 69], 2 [67, 70] used a non‐randomised trial design and 16 studies employed a cohort design [49, 50, 51, 54, 55, 58, 61, 62, 63, 66, 68, 71, 72, 73, 74, 75], 13 of which employed a single‐group repeated measures design with no comparison group [54, 55, 58, 61, 62, 63, 66, 68, 71, 72, 73, 74, 75]. The sample size of the included studies ranged from 28 to 11,092 participants [49, 55]. Most studies had a majority of male participants and the mean age was typically between 30 and 40 years.

**TABLE 1:** Summary of included studies.

Study, country	Study design (follow‐up length/study period)	Sample size	Age	Sex	Population/participant clinical characteristics	Co‐located service model	Comparison service model	Outcomes evaluated	Proportion of bias safeguards present and RRBS score
Bartels et al. (2004), USA	Multisite randomised controlled trial (6 months)	2022	Mean age = 73.5 years (SD ± 6.2). All participants were aged 65 years or older	74% male	69% had a primary diagnosis of depression (*n* = 1390); 20% met criteria for at‐risk drinking (*n* = 414); 3% met criteria for anxiety disorders; and 7% had a dual diagnosis (*n* = 148)	The integrated care modality had mental health and AOD treatment services—provided by licenced mental health/AOD treatment personnel—co‐located in a primary care setting	The referral model consisted of referrals by a primary care provider to mental health or AOD treatment offered in separate locations	Treatment engagement patterns: treatment engagement (yes/no); number of treatment appointments.	29/40; 73% RRBS: 91
Bhalla et al. (2021), USA	Retrospective cohort study (12 months)	11,092	Mean age = 56.5 years (SD ± 15.6)	92.7% male	Veterans treated in the integrated care clinic were more likely to have dual diagnosis (52.9%–20.9%) and be diagnosed with schizophrenia (19.2%–6.3%) and had a greater mean number of psychiatric diagnoses (2.12–1.76)	The integrated care clinic offered co‐located clinical care mental health care and AOD specialist treatment for serious mental illnesses and SUDs alongside primary care services, peer support, housing and criminal justice support and vocational rehabilitation	Care received through standard Veterans Health mental health and addiction clinics	Treatment engagement patterns: number of treatment appointments (total and by type—psychiatry, substance abuse, etc.). Health outcomes: psychotropic medication prescriptions. Health service use outcomes: inpatient treatment (by type—mental health medical etc.); ED attendances.	17/40; 43% RRBS: 53
Bond et al. (1991), USA	Prospective cohort design. Three sites had random assignment with a fourth not assigned at random (18 months)	Of 146 participants who were randomly assigned to treatment, 97 (66%) were retained in the study	Mean age = 31.5 years (SD not reported; range: 18–45)	79% male	All participants had chronic mental illnesses and diagnosis of SUD; 70% had a primary diagnosis of a schizophrenia spectrum disorder; and two‐thirds of the sample reported using alcohol and/or other drugs to relieve symptoms.	Across the treatment sites there was variation in implementation. Sites already specialising in AOD treatment hired a skilled and experienced mental health practitioner. Likewise, the sites already specialising in mental health treatment hired a skilled and experienced AOD specialist.	The control group received usual treatment at each of the community mental health centres participating in the study which had a group intervention focus using a psychoeducational approach.	Treatment engagement patterns: treatment engagement; treatment contact hours. Substance use patterns and harms: 3 alcohol and cannabis use frequency. Health outcomes: quality of life (measured by the Life Satisfaction Checklist). Health service use outcomes: number of hospitalisations; hospitalisation days.	21/40; 53% RRBS: 66
Bouchery et al. (2018), USA	Retrospective matched cohort study (6 years)	3489	40% over 64 years of age. Mean (SD) not reported	45% male	Schizophrenia was the most common psychiatric diagnosis (*n* = 942, 27%); 15% (*n* = 533) of the sample had dementia as their primary mental health diagnosis. No formal reporting on SUD prevalence.	Staff were organised into co‐located multidisciplinary care teams each consisting of a psychiatrist, a psychiatric nurse, Bachelor's level case managers, Master's level therapists, AOD specialists and medical assistants	Matched comparison group of Medicare clients at other mental health treatment facilities	Health service use outcomes: number of hospitalisations and ED attendances. Health economic outcomes: Medicare expenditures.	24/40; 60% RRBS: 75
Brooner et al. (2013), USA	Randomised controlled trial (1 year)	316	Mean age = 39.8 years (SD ± 0.5)	38% male	Opioid‐dependent participants with at least one comorbid psychiatric disorder. The most common diagnosis was major depression followed by PTSD. After opioid dependence, the most prevalent SUD was cocaine dependence	Participants were randomly assigned to receive a standard suite of psychiatric services (i.e., individual and group therapy, psychiatrist appointments, and pharmacotherapy) within an on‐site methadone program in an AOD treatment service	Community psychiatry on the same campus (at a different site than the intervention condition). Participants received parallel AOD treatment	Treatment engagement patterns: pharmacotherapy commencement; alcohol and other drug service utilisation. Health outcomes: psychological distress as measured by the Hopkins Severity Index.	32/40; 80% RRBS: 100
Clark et al. (1998), USA	Randomised controlled trial (3 years)	193	Mean age = 34 (SD not reported; range: 18–60)	74% male	54% of participants had a primary diagnosis of schizophrenia; 23% were diagnosed with schizoaffective disorder; and 24% met criteria for bipolar disorder	ACT teams were located within community mental health centres and provided direct AOD treatment, group therapy/support and had a clear team‐focus on dual diagnosis	SCM did not have a dual diagnosis team focus and did not provide direct AOD treatment. SCM also provided a less intensive service than ACT	Health economic outcomes: mental health inpatient and outpatient costs; general health inpatient and outpatient costs.	23/40; 58% RRBS: 72
Clausen et al. (2020), Norway	Prospective cohort study (2 years)	142	Mean age = 40 years (SD ± 8.7)	67% male	87% of participants had primary diagnoses of schizophrenia spectrum disorders. More than half (59%) of participants identified problematic substance use at entry.	12 multidisciplinary ACT teams across Norway providing mental health, AOD treatment, welfare and psychosocial services	None. Single‐group repeated measures design	Substance use patterns and harms: problematic substance use as measured by the AUDIT and the DUDIT. Health outcomes: psychiatric symptoms as measured by the BPRS—Expanded version. Level of functioning as measured by the Global Assessment of Functioning and the Practical and Social Functioning Scale. Quality of life as measured by the Manchester Short Assessment of Quality of Life.	27/40; 68% RRBS: 84
Cooper et al. (2010), USA	Prospective cohort study (1 year)	555	68% were aged between 26 and 45 years (mean age and SD not reported)	50% male	All participants reported having an Axis I or II mental disorder diagnosis and were assessed to have a SUD	IACT utilised assertive outreach delivered by a team consisting of AOD treatment counsellors, a psychiatrist, a nurse practitioner and several case managers with mental health and AOD treatment training.	None. Single‐group repeated measures design	Substance use patterns and harms: frequency of substance use. Health outcomes: psychiatric symptom severity measured using the BSI subscale scores. Health service use outcomes: ED presentations; mental health service utilisations.	21/40; 53% RRBS: 66
Drake et al. (1993), USA	Prospective cohort study (4 years)	18	Mean age = 38 years (SD ± 12.2)	67% male	All participants had diagnoses of co‐occurring schizophrenia and AUD (as per DSM‐III‐R)	All participants were treated in a dual diagnosis program and received assertive case management (at least weekly appointment), antipsychotic medication, housing support, and individual substance misuse counselling alongside mental health services	None. Single‐group repeated measures design	Substance use patterns and harms: remission of AUD (defined as more than 6 months of abstinence); length of remission	16/40; 40% RRBS: 50
Drake et al. (1998), USA	Randomised controlled trial (3 years)	203	Mean age = 34 years (SD ± 8.5; range: 18–60)	74% male	All participants had a DSM‐III‐R Axis I diagnosis of bipolar disorder, schizophrenia, or schizoaffective disorder, and an active SUD diagnosis within the past 6 months, and an absence of additional medical conditions or cognitive disability. 75% of the sample were diagnosed with schizophrenia or schizoaffective disorder and AUD (73%) was the most common SUD in the sample	Integrated and co‐located mental health and AOD treatment embedded in an ACT model which included dual‐disorder treatment groups, use of a stage‐wise dual‐diagnosis model and an exclusive team focus on dual disorders	Standard case management incorporated mental health and AOD treatment in a parallel referral model to services in separate locations	Substance use patterns and harms: abstinence from AOD. Health outcomes: quality of life. Health service use outcomes: inpatient hospital admissions.	31/40; 78% RRBS: 97
Drake et al. (2004), USA	Randomised control trial (3 years)	51	Mean age = 37.5 years (SD ± 9.6). Most patients were aged between 30 and 50 years	65% male	All patients had co‐occurring bipolar disorder and SUD, commonly misuse of alcohol, cannabis and/or cocaine	Integrated and co‐located mental health and AOD treatment embedded in an ACT model.	Standard case management incorporated mental health and AOD treatment in a parallel referral model to services in separate locations	Substance use patterns and harms: abstinence from AOD. Health outcomes: remission of psychiatric symptoms; quality of life. Health service use outcomes: days of inpatient services.	22/40; 55% RRBS: 69
Drake et al. (2016), USA	Prospective cohort study, a secondary analysis of an RCT (7 years—4 years follow‐up post‐RCT)	150	Mean age = 36.7 years (SD = 7.6)	75% male	All participants were diagnosed with co‐occurring schizophrenia‐spectrum (schizophrenia, schizoaffective disorder or bipolar disorder) and SUD	Multidisciplinary teams provided integrated and co‐located mental health and AOD treatment, motivational interviewing and dual diagnosis recovery groups	None. Single‐group repeated measures design	Substance use patterns and harms: remission of SUDs. Health outcomes: absence of psychiatric symptoms; quality of life. Health service use outcomes: hospital admission days.	23/40; 58% RRBS: 72
Fletcher et al. (2008), USA	Randomised controlled trial (2.5 years)	191	Mean age = 40 years (SD ± 9.13; range 18–66)	80% male	All participants were homeless, had a severe mental illness (schizophrenia, MDD, bipolar, schizoaffective or delusional disorder) and DSM‐IV SUD; 42% had both drug and alcohol disorders, 40% had an AUD only and 18% had a drug use disorder only. Cocaine, predominantly crack cocaine, was the drug most frequently used (29%) followed by cannabis (22%)	The IACT team included mental health and AOD treatment specialist staff and provided co‐located outpatient AOD use counselling and bi‐weekly treatment groups	The ACTO team referred clients to other community providers for outpatient AOD treatment services and/or to 12‐step groups. The standard care group were provided with information regarding local services that provided mental health or drug and alcohol treatment and were assisted to make initial contact with these services	Substance use patterns and harms: substance misuse rating based on a five‐point scale from ‘no use’ to ‘severe use and related problems’. Health outcomes: consumer satisfaction; psychiatric symptoms.	25/40; 63% RRBS: 78
Frisman et al. (2009), USA	Randomised controlled trial (3 years)	124	Mean age = 36.8 years (SD ± 7.9)	72% male	All participants were diagnosed with co‐occurring schizophrenia‐spectrum disorders (schizophrenia, schizoaffective disorder, or bipolar disorder) and SUD; 36 individuals had Anti‐Social Personality Disorder	Multidisciplinary teams provided integrated and co‐located mental health and AOD treatments, motivational interviewing, and dual diagnosis recovery groups	Standard case management incorporated and integrated mental health and AOD treatment in a parallel referral model to services in separate locations	Substance use patterns and harms: alcohol use frequency.	21/40; 53% RRBS: 66
Granholm et al. (2003), USA	Retrospective cohort study (not reported)	44	Mean age = 42 years (SD ± 8.2)	89% male	All patients had a SUD and an independent Axis I mental illness	Co‐located brief intervention (maximum: 6 months) in a dual diagnosis service with a multidisciplinary treatment team comprised of a psychiatrist with addiction training, a psychologist, and a social worker alongside advanced trainees from each discipline (senior residents or interns)	None. Retrospective pre‐post evaluation design	Health service use outcomes: psychiatric hospitalisations; inpatient days for SUD.	21/40; 53% RRBS: 66
Holdcraft et al. (2002), USA	Prospective cohort study (2 years)	20	Mean age = 39.3 years (SD ± 10.0)	100% female	All participants had been diagnosed with a major mental illness. Not all women in the program had a dual diagnosis; a SUD was not required for admission	Dual diagnosis service staff consisted of a psychologist, one advanced psychiatry resident, one master's level social worker, and a psychiatrist who performed individual/group therapy and medication management. The program included a CBT skills group (focusing on substance misuse).	None. Single‐group repeated measures design	Substance use patterns and harms: length of abstinence from AOD. Health outcomes: Quality of life. Health service use outcomes: number of psychiatric hospitalisations.	19/40; 48% RRBS: 59
Judd et al. (2003), USA	Prospective cohort study (3 years)	126	Mean age = 38 years (SD: not reported)	54% male	All participants had a DSM‐IV Axis I psychiatric disorder and SUD	Co‐located dual diagnosis service comprised of psychiatric residents, a master's level social workers who served as care coordinator and therapist, a community aide and a recovery counsellor	None. Single‐group repeated measures design	Substance use patterns and harms: Kennedy Axis‐5 scales; Basis‐32 Impulsive/Addictive scale, Addiction Severity Index. Health outcomes: SF‐12 Mental Health Scale; The Basis‐32 Psychosis, Depression and Anxiety scale. Health economic outcomes: Addiction treatment costs. Physical health costs. mental health treatment costs. hospitalisation costs.	24/40; 60% RRBS: 75
Kidorf et al. (2013), USA	Randomised controlled trial (3 months)	125	Mean age = 39.1 years (SD: not reported)	46% male	Major depression, PTSD, and panic disorder were the most prevalent DSM‐IV Axis I psychiatric disorders: 44% of urine samples collected at baseline tested positive for at least one illicit drug and >40% of participants were diagnosed with antisocial personality disorder	Methadone maintenance in a community‐based AOD treatment program received integrated and co‐located psychiatric care that included individual and group mental health counselling sessions, psychiatrist appointments, and consistent access to prescribed psychiatric medications. Received vouchers as reinforcement for attending treatment.	Standard on‐site care with an identical set of services offered in the program, however, no vouchers were provided for attendance.	Substance use patterns and harms: opioid‐positive and sedative‐positive urine drug screens. Health outcomes: symptom Checklist—Global Severity Index (SCL‐90‐R GSI) scores over time.	30/40; 75% RRBS: 94
Lee et al. (2009), USA	Randomised controlled trial (6 months)	34	Mean age = 72.9 years (SD ± 6.8). All participants were aged 65 years or older	59% male	Older persons who exhibited significant psychological distress; had suicidal ideation, and reported at‐risk alcohol consumption defined as drinking more than 12 drinks per week for women, more than 14 drinks per week for men, or 4+ drinks, 4+ times in the previous 3 months (i.e., binge drinking)	A primary care clinic with program staff including co‐located primary care doctors, nurses, nurse practitioners, physicians' assistants, social workers, AOD counsellors, psychologists, and a psychiatrist	A 12‐step program within a community centre that provided other activity‐ and social‐based services to older adults with low‐income	Treatment engagement patterns: total time in treatment; time from initial assessment to treatment. Substance use patterns and harms: average number of drinks per week; past month binge drinking episodes.	24/40; 60% RRBS: 75
Logan et al. (2019), USA	Retrospective cohort study using clinical record extraction (3 months)	101	Mean age = 43 years (SD ± 12.8)	55% male	Participants met criteria for moderate or severe opioid use disorders, 75% also had at least one other SUD (41% alcohol, 39% methamphetamine); 77% of participants were diagnosed with a comorbid mental illness, with depression and anxiety being most common.	Alcohol and other drug treatment and psychological specialist services co‐located within a rural primary care setting which offered behavioural health assessment and therapy, prescribed medication treatment, and case management services in the same location	None. Single‐group retrospective clinical record extraction	Treatment engagement patterns: buprenorphine program retention. Health outcomes: depressive symptoms.	20/40; 50% RRBS: 63
Mangrum et al. (2006), USA	Non‐randomised trial (1 year)	216	Mean age = 37 years (SD: not reported)	49% male	The most common principal diagnoses in both groups were major depression, schizophrenia, and bipolar disorder. Primary alcohol or drug use disorders were prevalent in 2% and 7% of participants, respectively. The prevalence of dual diagnosis was not reported.	A dual diagnosis treatment team was established at all study sites to provide case management and treatment oversight for clients with co‐occurring mental illness and SUD. Each program provided individual therapy, and specialised group therapy tailored to dual diagnosis issues.	Parallel provision of AOD treatment and mental healthcare by separate clinics. No service provision coordination was provided between the treatment programs and there was no centralised case management component	Health service use outcomes: incidence of all‐cause inpatient hospital admissions; number of psychiatric hospitalisation days.	21/40; 53% RRBS: 66
McFall et al. (2006), USA	Prospective cohort study (9 months)	107	Mean age = 51 years (SD ± 6.8)	92% male	Participants were recruited from two PTSD clinics and included if they met DSM‐IV criteria for PTSD and were tobacco dependent defined as smoking 10 or more cigarettes per day and reported a willingness to receive smoking cessation treatment. Participants had a mean of approximately 2 co‐occurring Axis I disorders	Participants received psychotropic medications and supportive psychotherapy for PTSD from case managers responsible for coordinating their mental health care. The co‐located multidisciplinary team included: a psychiatrist, six psychologists, two AOD treatment specialists, three nurses, two social workers, one physician assistant, and four behavioural health technicians	None. Single‐group repeated measures design	Substance use patterns and harms: abstinence from smoking verified by carbon monoxide readings; number of cigarettes smoked per day.	25/40; 63% RRBS: 78
Morse et al. (2006), USA	Randomised controlled trial (2 years)	149	Mean age = 40 years (SD: not reported; range: 18–66)	80% male	All participants had a severe mental illness (operationalised as schizophrenia, atypical psychosis, bipolar disorder, recurrent major depression, schizoaffective disorder or delusional disorder) and had a DSM‐IV SUD	Participants were randomly assigned to one of three treatment conditions: IACT, ACTO or standard care (control). The IACT had an AOD treatment specialist and provided substance use treatment directly, co‐located as part of the ACT within a mental healthcare agency	The ACTO team referred clients to other community providers for AO treatment and/or to 12‐step groups. Participants in the standard care control condition were provided a list of community mental health and AOD treatment agencies with no further support to engage with these services.	Treatment engagement patterns: number of treatment service contacts. Health economic outcomes: program costs; other outpatient service costs; inpatient treatment costs.	24/40; 60% RRBS: 75
Morse et al. (2008), USA	Non‐randomised trial (18 months)	270	Mean age = 40 years (SD ± 9; range 18–66)	76% male	All participants had a severe mental illness (bipolar disorder, schizophrenia, recurrent major depression, schizoaffective disorder, atypical psychosis, or delusional disorder) and had a DSM‐IV SUD	Participants were assigned to one of four treatment conditions: NIACT, IACT, ACTO or standard care (control). NIACT had all the elements of IACT, but various program components and operations were manipulated (e.g., conducting treatment‐planning sessions twice a week that focused on substance use issues and increasing the number of AOD specialists from one to two per team)	NIACT was compared to the three treatments outlined by Morse et al. (2006)	Treatment engagement patterns: treatment fidelity; number of treatment service contacts. Substance use patterns and harms: frequency of drug use. Health outcomes: psychiatric symptoms measured by the BPRS.	20/40; 50% RRBS: 63
Neufeld et al. (2010), USA	Retrospective cohort study using clinical record extraction (8 weeks)	81	Mean age = 43.5 years (SD: not reported)	43% male	All participants had current opioid dependence and met Centre for Substance Abuse Treatment criteria for long‐term use of methadone and other opioid–agonist medications; 41% of participants reported at least one co‐occurring mental illness	Co‐located in the same site, the Community Access to Specialised Treatment team consisted of psychiatrists, clinical psychologists and senior certified AOD associate counsellors.	None. Single‐group retrospective clinical record extraction	Treatment engagement patterns: proportion of program completions; average number of days in program; number of sessions attended; medications commenced. Health outcomes: psychiatric evaluation; newly diagnosed psychiatric conditions.	20/40; 50% RRBS: 63
Rasch et al. (2013), USA	Prospective cohort study (6 months)	426	Mean age = 34.5 years (SD ± 10.3)	63% male	All participants were diagnosed with a co‐occurring psychiatric and SUD.	The treatment delivery staff included two master's level therapists, an intake worker, a nurse (a registered nurse with expertise in HIV and AOD treatment) and a psychiatrist co‐located in the same service site	None. Single‐group repeated measures design	Treatment engagement patterns: average length of stay in program. Substance use patterns and harms: substance use as measured by the *Government Performance and Results Act* Instrument. Health outcomes: quality of life and life satisfaction measured with the California Quality of Life Survey; mental health symptoms as measured on the BSI.	22/40; 55% RRBS: 69
Walter et al. (2022), USA	Prospective cohort study (1 year)	107	Mean age = 44 years (SD ± 9.6)	69% male	At baseline, 47% of participants had moderate or severe depressive symptoms, and 43% had moderate or severe anxiety symptoms. About 22% of participants reported illicit drug use in the past 30 days	Co‐located and integrated dual diagnosis treatment and primary health care provided by a multi‐disciplinary team with experience providing care within the diverse Latinx culture and language	None. Single‐group repeated measures design	Substance use patterns and harms: past 30 days illicit drug use. Health outcomes: depressive and anxiety symptoms. Health service use outcomes: ED presentations in the past 30 days.	24/40; 60% RRBS: 75
Xie et al. (2005), USA	Prospective cohort study (3 years)	152	Mean age = 32 years (SD ± 7.2)	78% male	Patients with co‐occurring schizophrenia (schizophrenia or schizoaffective disorder) and SUD	Participants were randomly assigned ACT or standard case management, both of which were provided at the same location and entailed integrated mental health care and AOD specialist treatments	None. Single‐group repeated measures design. (Standard case management and assertive treatment groups were analysed together).	Substance use patterns and harms: substance use frequency and severity (measured by days of use and the Addiction Severity Index). Health outcomes: psychiatric symptoms. quality of life and life satisfaction. Health service use outcomes: inpatient hospital admissions.	27/40; 68% RRBS: 84

Abbreviations: ACT, Assertive Community Treatment; ACTO, Assertive Community Treatment only; AOD, alcohol and other drugs; AUD, alcohol use disorder; AUDIT, Alcohol Use Disorder Identification Test; BPRS, Brief Psychiatric Rating Scale; BSI, Brief Symptom Inventory; DSM‐III‐R, Diagnostic and Statistical Manual of Mental Disorders, Third Edition, Revised; DSM‐IV; Diagnostic and Statistical Manual of Mental Disorders, Fourth Edition; DUDIT, Drug Use Disorder Identification Test; ED, emergency department; IACT, Integrated Assertive Community Treatment; MDD, major depressive disorder; NIACT, New Integrated Assertive Community Treatment; PTSD, post‐traumatic stress disorder; RRBS, relative risk of bias score; RCT, randomised controlled testing; SCM, standard case management; SUD, substance use disorder.

Substance use disorder and mental illness profile among participants with dual diagnosis varied across the included studies. Eight of 28 studies [51, 53, 55, 56, 58, 67, 74, 75] included participants who had either a primary or ‘predominant’ diagnosis of a schizophrenia spectrum disorder (schizophrenia or schizoaffective disorder). Seven of 28 studies [48, 49, 52, 64, 66, 67, 73] reported high proportions of participants with depressive disorders. Three of the 28 studies [52, 66, 71] only included participants meeting criteria for opioid use disorder. Another 4 of the 28 studies [49, 56, 59, 65] reported high rates (22–82%) of alcohol dependence. Cocaine [52, 54, 57, 59, 72] and cannabis [54, 57, 59, 72] dependence or misuse were also common among participants in the included studies.

### 3.2 Models of service co‐location

All included studies investigated the efficacy and outcomes of integrated care models with co‐located mental health care and AOD specialist treatment (Table 1). Twenty‐two of the 28 included studies examined co‐located multidisciplinary teams [48, 50, 51, 53, 54, 55, 56, 57, 58, 60, 61, 62, 63, 65, 66, 67, 69, 70, 71, 72, 73, 75], often in the context of Assertive Community Treatment (ACT) models [53, 54, 56, 57, 69, 70, 75], which frequently included case managers, medical nurses, psychiatric nurses, psychiatrists and counsellors or psychologists in addition to AOD specialists. Of the remaining six studies, three [49, 59, 74] were dedicated dual diagnosis services with mental health care and AOD treatment specialists providing care in the same service location. One study [68] was a model where an AOD specialist was embedded in a mental health‐care service location, and two [52, 64] were models in which a mental health specialist was embedded in an AOD treatment service location.

### 3.3 Treatment engagement patterns

Seven out of 28 studies [48, 49, 50, 52, 65, 69, 70] quantified the relationship between co‐located mental health and AOD specialist care, and treatment engagement patterns (Table S8). All seven studies reported a positive association between co‐located mental health care and AOD specialist treatment, and rates of treatment engagement. For example, Bartels et al. found that those in the co‐located and integrated treatment model were 22% more likely to engage in treatment, compared with those assigned to an enhanced referral model where participants were referred to a specialist mental health or AOD treatment service in a separate location nearby [48]. Another study observed that participants receiving on‐site and integrated psychiatric and AOD treatment specialist care were more likely to initiate psychiatric care, remained in treatment longer, and attended more appointments than those receiving off‐site and non‐integrated AOD treatment and psychiatric care [52].

Three additional studies [66, 71, 72] reported relatively high rates of engagement (>60%) and completion (>40%) over the study periods. However, these studies did not compare these rates to a control condition or an appropriate baseline measure.

### 3.4 Substance use patterns and harms

Eighteen of the 28 studies [50, 54, 55, 56, 57, 58, 59, 60, 62, 63, 64, 65, 68, 70, 72, 73, 74, 75] examined the relationship between co‐located mental health and AOD specialist care and substance use patterns and/or harms. Nine of the 18 studies [54, 55, 60, 63, 65, 70, 72, 73, 74] reported statistically significant reductions in substance use frequency, severity and/or related harms, particularly for the use of tobacco, alcohol, cocaine, cannabis and opioids, among participants who received co‐located treatment. Six of these nine studies [54, 55, 63, 72, 73, 74] reported these reductions over time in a repeated measures design without a comparison condition. Furthermore, two of these nine studies [63, 65] found no difference on at least one valid screening tool for substance use severity and/or harms (i.e., the Addiction Severity Index or the Short Michigan Alcohol Screening Test—Geriatric Version). Also, the decrease in illicit drug use from baseline to follow‐up reported by Walter et al. did not remain statistically significant after adjusting for sociodemographic covariates [73]. A further 4 of the 18 studies [50, 56, 59, 64] that assessed substance use patterns and/or harms found no correlation between co‐located mental health and AOD specialist treatment and substance use patterns and/or harms.

Eight of the 18 studies [54, 55, 57, 58, 62, 68, 74, 75] found a positive correlation between co‐located mental health care and AOD specialist treatment, and abstinence from substance use or remission from substance use disorder. For example, Drake et al. observed that 61% of patients with a dual diagnosis of schizophrenia and alcohol use disorder were in remission (defined as six or more months with no evidence of harmful alcohol use) after receiving 4 years of co‐located treatment [55]. Additionally, McFall et al. observed a positive correlation between time in treatment and likelihood of achieving abstinence from cigarette smoking when smoking cessation was co‐located and integrated into community mental health support [68]. Six of these eight studies [54, 55, 58, 62, 68, 74] reported these positive correlations over time in a repeated measures design without a comparison group. A further 2 of the 18 studies did not find a difference in abstinence or remission between co‐located mental health care and AOD specialist treatment and a comparison condition [56, 64].

### 3.5 Health outcomes

Seventeen out of 28 studies reported a relationship between co‐located care and at least one health outcome (Table S8) [50, 52, 54, 56, 57, 58, 59, 62, 63, 64, 66, 70, 71, 72, 73, 74, 75]. Ten of these 17 studies [52, 54, 58, 63, 64, 66, 72, 73, 74, 75] observed significant improvements in the mental health symptom severity of participants receiving co‐located mental health and AOD specialist treatment. For example, Judd et al. [63] found a positive correlation between time engaged in treatment and improvement in mental health as measured by the Kennedy Axis‐5 scales, the SF‐12 Mental Health Component Scale and the Basis‐32 Psychosis, Depression and Anxiety scale (*p* < 0.05). These improvements were observed after 6 months in treatment and were sustained throughout 3 years of follow‐up [63]. Similarly, Clausen et al. observed improvements in negative psychotic symptoms and symptoms of agitation, mania, anxiety and depression as measured using the Brief Psychiatric Rating Scale in those receiving co‐located ACT [75]. However, 8 of these 10 studies [54, 58, 63, 66, 72, 73, 74, 75] did not have a comparison condition. An additional 2 of the 17 studies [59, 70] measured mental health symptom severity and reported no effect due to co‐located mental health and AOD service provision when compared to a control condition.

Five of the 17 studies examining health outcomes [56, 57, 58, 72, 74] reported a positive association between mental health and AOD specialist treatment and quality of life. For example, Rasch et al. observed statistically significant improvements in five of seven quality of life areas assessed, and overall general life satisfaction improved after 6 months [72]. Similarly, Drake et al. found significant improvements in quality of life over time for both the co‐located and a standard case management group on the Quality‐of‐Life Interview [56]. The magnitude of the quality‐of‐life improvement was greater for participants receiving co‐located care than for those in the standard case management group (17% vs. 7% relative improvement over 3 years) [56]. Three additional studies reported no difference in quality of life over follow‐up in a repeated measures design with no comparison condition [50, 62, 75].

One of the 17 studies examining health outcomes reported no reduction in the number of suicide attempts over 6 months of receiving co‐located mental health and AOD specialist care compared to pre‐treatment (baseline) rates [72]. No other study reported morbidity, mortality rates or rates of other health conditions beyond substance use patterns and psychiatric symptoms.

### 3.6 Health service use outcomes

Twelve out of 28 studies [49, 50, 51, 54, 56, 57, 58, 61, 62, 67, 73, 74] reported at least one health service use outcome (Table S8). Eight of these 12 studies [50, 51, 56, 57, 58, 61, 62, 67] reported decreases in inpatient hospital admissions and/or hospital admission days for participants who received co‐located mental health care and AOD specialist treatment. For example, Drake et al. [57] observed that participants who received integrated, co‐located care decreased their inpatient service utilisation steadily over 3 years. Similarly, Bond et al. found that participants receiving co‐located ACT incorporating mental health care and AOD specialist treatment had fewer hospitalisation days 18 months after treatment initiation [50]. However, one study observed no difference in inpatient hospital service use for participants with schizophrenia and co‐occurring substance use disorder who received co‐located assertive mental health care and AOD specialist treatment compared to a parallel referral model [74]. In this study, co‐located care was associated with increased mental health outpatient service usage over the follow‐up period whereby participants increased outpatient case management contacts and medication visits specifically [74]. Conversely, Bhalla et al. observed that veterans receiving co‐located care were more likely to attend general psychiatry appointments, attend outpatient AOD treatment visits and receive inpatient mental health treatment than those receiving standard care [49]. Three of these eight studies [58, 61, 62] reported changes in service utilisation over time in a repeated measure design without a comparison condition.

Three of the 12 studies [51, 54, 73] observed correlations between co‐located mental health and AOD specialist treatment and decreased rates of emergency department presentations. For example, in a repeated measures cohort design, Walter et al. found that participants receiving integrated care provided by a co‐located multi‐disciplinary team including mental health care and AOD treatment specialists were 60% less likely at 12‐month follow‐up to have visited an emergency department in the past 30 days, compared to the 30 days prior to treatment enrolment [73].

Cooper et al. observed that inpatient substance use treatment declined among participants receiving integrated ACT (comprised of co‐located outpatient mental health and AOD specialist treatment complemented by assertive outreach, motivational enhancement, counselling, vocational rehabilitation and peer support) [54]. Furthermore, participants experienced significant decreases in inpatient mental health and inpatient (residential) AOD treatment over 12 months of follow‐up [54].

### 3.7 Health economic outcomes

Four out of 28 studies [51, 53, 63, 69] investigated and observed cost reductions associated with co‐located and integrated mental healthcare and AOD specialist treatment (Table S8). Bouchery et al. estimated that during the first 2.5 years of program implementation, Medicare expenditures decreased on average by US $266 per enrolled beneficiary per month for the intervention group versus the comparison group (*p* < 0.01) [51]. Similarly, Clark et al. noted that participants receiving co‐located assertive integrated community treatment had lower costs in mental health inpatient treatment (US $27,604 vs. US $34,006) and general health inpatient treatment (US $645 vs. US $1345) compared with those receiving standard case management treatment at 3 years follow‐up [53]. Likewise, Judd et al. estimated a decrease of US $1010 in the 2 years after treatment per enrolled patient in the cost of alcohol and drug treatment services, but found an increase in the average per patient costs for mental health treatments of US $1412 [63]. However, an overall cost reduction in the use of acute and subacute levels of mental healthcare was found (i.e., emergency department presentations and inpatient psychiatric hospitalisation). The authors concluded that increased mental health‐care costs were related to greater use of outpatient mental health services, supported living situations and skilled nursing facilities [63].

By contrast, Morse et al. observed reductions in outpatient costs for those who received co‐located care but there were no differences in inpatient costs when two forms of co‐located ACT were compared with a standard care model involving referral information for specialist mental health or AOD treatment services [69].

### 3.8 Quality assessment

The proportion of bias safeguards present and the relative risk of bias score of each study on the MASTER scale is summarised in Table 1 and presented in Table S9. The proportion of bias safeguards present in the included studies ranged from 40% to 80% with a median of 58% (interquartile range: 53–63%). Thirteen studies [48, 51, 52, 56, 59, 63, 64, 65, 68, 69, 73, 74, 75] scored above the median relative risk of bias score and were considered to have low risk of bias (i.e., high quality).

Sensitivity analyses broadly supported our primary findings (see Data S1). However, a high proportion of studies that reported a correlation between co‐located mental health and AOD specialist treatment and acute care use had a high risk of bias.

## 4 DISCUSSION

Despite approximately two decades of recommendations to implement integrated mental health care and AOD specialist treatment models [76, 77, 78, 79, 80], our review highlights a lack of high‐quality evidence on the impact of co‐location of mental health care and AOD specialist services on the health outcomes these integrated models aim to achieve. Most co‐located service models differed from the comparison models of care beyond co‐location, including differing levels of care coordination, integration of service provider processes and clinical service team composition. Therefore, as we could not isolate the independent effect of physical co‐location, our findings are most accurately interpreted as outcomes related to integrated care models that include co‐location of mental health and AOD specialist treatment. We found evidence that integrated care that includes co‐located mental health and AOD specialist treatment is associated with improvements in treatment engagement, reductions in substance use and related harms, reductions in mental health symptom severity, decreases in emergency department presentations, hospital admissions and stay length, and reductions in health system expenditure. However, the evidence was heterogeneous, relatively limited in scope, based almost exclusively on samples from the United States, and many studies had a relatively high risk of bias.

Our findings parallel those from a broader review of psychosocial interventions provided to people with dual diagnosis in which the authors concluded that substandard methods, poorly reported intervention methodology, and a high risk of bias in the included studies limited their ability to establish the effectiveness of integrated models of care compared to standard care [81]. Another prior review reported a similar lack of evidence to establish health outcomes related to co‐located and integrated youth mental health care and AOD service hubs, and the key attributes of these hubs that predict their effectiveness [82].

We found consistent evidence that integrated care that included co‐located mental health care and AOD specialist treatment was positively correlated with improved patterns of treatment engagement and remission/abstinence from substance use. Prior research has highlighted multiple barriers to accessing community‐based mental health care and AOD treatment for people with dual diagnosis [25, 83, 84]. Structural barriers, including service availability/logistical problems, a lack of provider knowledge of referral options, staff awareness and capacity for identification of co‐occurring disorders, and a lack of sharing information and administrative delays, are consistently reported as impediments to accessing mental health and/or AOD treatment for people with dual diagnosis [25, 83]. Furthermore, once engaged with mental health care or AOD treatment, there is comparatively poor retention in these services among people with dual diagnosis [85], particularly if their co‐occurring disorder is not identified and addressed in treatment [86]. Structural issues stemming from the historical and largely artificial separation between mental health care and AOD treatment systems may further contribute to poor retention in treatment [87, 88]. In this context, and considering our findings, there is face validity for situating mental health and AOD specialist services in the same location to address these common structural barriers to the engagement and retention of people with dual diagnosis in treatment.

Increased symptom severity and complexity associated with dual diagnosis often presents challenges for clinicians in AOD treatment or mental health‐care settings [89, 90, 91, 92]. If not identified and managed adequately, the co‐occurrence of mental illness can often aggravate and reinforce symptoms of substance use disorder and vice versa [93, 94]. Accordingly, current guidelines recommend treating and managing both conditions simultaneously [77]. Integrated models of care, which address chronic physical health conditions and mental illness concurrently, achieve better physical and mental health outcomes regardless of which condition is the primary target of the treatment [95]. Similarly, our findings provide evidence that integrated care that includes co‐located mental health and AOD specialist treatment likely improves substance use patterns and related harms and mental health symptom severity, both over time in treatment and when compared to standard referral‐based models of care. However, this assertion is based on relatively few studies with moderate to high risk of bias, thus caution should be applied when interpreting these findings.

There is good evidence that dual diagnosis is associated with substantial morbidity and mortality, beyond what is observed among people with mental illness or substance use disorder alone [10, 14, 15, 16, 17]. Consistent with other studies that have examined health outcomes associated with integrated multidisciplinary models of care [96], we found modest evidence that integrated care that included co‐located mental health and AOD specialist treatment was correlated with reduced mental health symptom severity and improved quality of life. However, our findings highlight that we know relatively little about the specific impact of mental health and AOD specialist treatment co‐location on health outcomes for people with dual diagnosis.

Prior research has established a positive association between dual diagnosis and rates of acute care use [97]. Co‐located primary care and mental health care has been associated with decreased rates of emergency department presentations, inpatient hospital admissions and hospital bed days for people with moderate to severe mental illness [98]. A subsequent systematic review and meta‐analysis that examined the effect of co‐located primary and any specialist care on the rate of hospitalisation did not find a significant pooled association from the three included studies which measured this outcome [96]. In contrast, among the studies in our review that quantified the impact of co‐located mental health and AOD specialist treatment on rates of acute care use, there was consistent evidence that co‐located care was associated with reduced rates of hospitalisation, hospital bed days, and emergency department presentations to a lesser extent, although the quality of this evidence was low. Given that acute health‐care use, especially inpatient care, is a major driver of health system costs [99], our findings suggest that integrated care that includes co‐located mental health and AOD specialist treatment may have the potential to improve health and reduce health system expenditure. However, given that no study in our review conducted a formal cost‐effectiveness analysis and some studies observed increased contacts with inpatient, outpatient or secondary services, it is currently unclear if co‐located mental health and AOD specialist treatment models are cost‐effective.

### 4.1 Implications for policy and practice

Recommendations to integrate mental health‐care and AOD services, and provide a person‐centred model of care for those who experience dual diagnosis [77, 100] have resulted in considerable investment to build capacity and implement collaborative multidisciplinary models with varying degrees of care coordination, service integration and co‐location [101, 102]. Broadly, the findings of our review indicate that, although integrated care that includes co‐located mental healthcare and AOD specialist treatment is associated with some positive outcomes for those with dual diagnosis, the evidence is still limited, and it is currently unclear which models of integrated and co‐located care achieve the best health outcomes and value for money.

Despite common overlap in their implementation, the elements of care coordination, co‐location and integration of service provider processes have been conceptually differentiated in integrated care models [42, 103]. For example, two services can have their processes integrated while not being co‐located and conversely, services can share the same clientele and location and not integrate their service processes or care provision to their clients [103]. Although co‐location is not sufficient to ensure service integration [42], it has been established as an effective strategy to increase the integration of service processes [104]. We found that co‐location and service integration were not clearly defined, often not described in detail, were conflated, or not measured separately in the peer‐reviewed literature examining the health impact of co‐located mental health and AOD specialist treatment. Our findings suggest that integrated care models that include co‐location of mental healthcare and AOD specialist treatment improves health outcomes to a greater extent than parallel (e.g., referral based) care coordination. However, as we could not separate the effect of co‐location from care coordination and integration of service provider processes, these definitional and measurement issues in the current literature limit our understanding of independent impact of co‐location of mental health care and AOD specialist treatment on health outcomes for people with dual diagnosis.

When taken together, we found a limited body of evidence that integrated care that includes co‐located mental health and AOD specialist treatment is associated with improved substance use treatment outcomes, reduced mental health symptom severity, and decreased rates of acute care use. Therefore, our findings provide provisional support for the evaluation of, and potential investment in, co‐located mental health care and AOD specialist service delivery. However, we identified little high quality, peer‐reviewed evidence to guide stakeholders and policymakers tasked with the implementation of these collaborative mental health and AOD treatment models. Without investment in rigorous evaluation, evidence in this area will remain insufficient to establish the effectiveness of co‐located mental health care and AOD treatment and guide the optimisation of the efficacy of these integrated service models. Currently, evidence‐based policy in this area can only be considered aspirational.

### 4.2 Limitations

To our knowledge, this is the first study to synthesise the peer‐reviewed literature on the relationship between integrated care that includes co‐located mental health and AOD specialist treatment, and treatment engagement and substance use, health outcomes, health service use, and health economic outcomes. However, our findings have some limitations. We could not isolate the independent effect of physical co‐location, therefore our findings our limited to the impact that co‐location of mental health and AOD specialist treatment has within broader integrated care models. All but one of the included studies was conducted in the United States. Given that the US health system differs substantially from many other countries, the evidence generated in the United States may not be generalisable to countries with different health system composition. Men were disproportionately represented in the studies included in our review. As such, the applicability of our results to women with a dual diagnosis is less clear. Approximately two‐thirds of the included studies were conducted prior to 2010 and therefore our findings may not be generalisable to current substance use patterns and treatment approaches for those with dual diagnosis. Additionally, the dearth of studies on long‐term health outcomes using administrative morbidity or mortality data is a significant limitation of the current evidence base in this area. We also limited our search strategy to articles published in English. Thus, we may not have identified relevant articles in other languages.

## 5 CONCLUSION

Integrated care that includes co‐located mental health and AOD specialist treatment may be associated with better treatment engagement and substance use outcomes, improvements in mental health symptoms and quality of life, reduced emergency department presentations and hospitalisations, and decreased health system expenditure for people with a dual diagnosis of mental illness and substance use disorder. The quality of studies included in this review was relatively low and did not allow us to disaggregate the independent effect of physical co‐location from care coordination or integration of service processes. Therefore, to inform policy and optimise practice there is an urgent need for further high‐quality longitudinal research and well‐designed trials that examines the independent effect of co‐location as a common component of integrated models of care. However, the available evidence indicates that integrated care that includes co‐location of mental healthcare and AOD specialist treatment may yield health and economic benefits.

## AUTHOR CONTRIBUTIONS

Clare Glover‐Wright and Jesse T. Young developed and wrote the original manuscript. Kym Coupe, Alexander Charles Campbell, Claire Keen, Patrick Lawrence, Stuart A Kinner and Jesse T. Young contributed significantly to drafting and editing the manuscript. All authors approved the final manuscript. Apart from the funding bodies listed above, this research received no specific grant from any funding agency in the public, commercial or not‐for‐profit sectors. The funding source had no additional role in the research design; data collection, analysis or interpretation; the writing of the manuscript; or the decision to submit the article for publication.

## CONFLICT OF INTEREST

Other than the funding sources listed above, the authors declare no potential conflicts of interest with respect to the research, authorship and/or publication of this article.

## Supporting information


**Data S1:** Supporting information
