# Supplementary material for: Health outcomes and service use patterns associated with co‐located outpatient mental health care and alcohol and other drug specialist treatment: A systematic review
Source: Drug Alcohol Rev. 2023 Apr 4;42(5):1195–219. doi: 10.1111/dar.13651 (PMC10946517; doi:10.1111/dar.13651)
Supplement: Supplementary file 1 — Data S1: Supporting information [file DAR-42-1195-s001.docx]

**Health outcomes and service use patterns associated with co-located outpatient mental healthcare and alcohol and other drug specialist treatment: A systematic review**

**SUPPORTING INFORMATION**

## ***Results - Sensitivity analysis***

## When we restricted our analyses to studies that a low risk of bias we found that, of the seven studies that reported a positive association between co-located mental health and alcohol and other drug (AOD) specialist treatment and rates of treatment engagement, four had a low risk of bias [1-4]. Of the nine studies that reported reductions in substance use patterns and related harms, four [3,5-7] had a low risk of bias. By contrast, three of the four studies [8-10] that assessed substance use patterns and/or harms found no correlation with co-located mental health and AOD specialist treatment, and had a low risk of bias. Three of the eight studies [7,11,12] that found a positive correlation between co-located mental health and AOD specialist treatment and abstinence from substance use or remission from substance use disorder had a relatively low risk of bias.

## Six of the 10 studies [2,5-7,10,12] that found significant improvements in the mental health symptom severity of participants receiving co-located mental health and AOD specialist treatment had a low risk of bias. Of the two studies that measured mental health symptom severity and reported no effect due to co-located mental health and AOD service provision, one was assessed to have a low risk of bias [9]. Two [7,8] of five studies that found a positive association between mental health and AOD specialist treatment and participant quality of life were assessed to have a low risk of bias. Neither of the two additional studies which evaluated and found no correlation between co-located mental health and AOD specialist treatment and quality of life had a low risk of bias [13,14].

## Three of the ten studies [6,8,15] that found an association between co-located mental health and AOD specialist treatment and decreased emergency department presentations, inpatient hospital admissions and/or number of hospital days were assessed to have a low risk of bias. Three of the four studies [4,5,15] that observed cost reductions attributable to co-located and integrated treatment had a relatively low risk of bias.

**Table S1.** **Study characteristic definitions according to the PICO framework**

| **Domain** | **Definition** |
| --- | --- |
| Population | People with a dual diagnosis of mental illness and substance use disorder, both with and without evidence of a formal diagnosis. Consistent with prior research,[16] we operationally defined dual diagnosis according to the International Classification of Diseases, 10^th^ edition, Australian Modification diagnostic codes for mental illness (F00-F09; F20-F99) and substance use disorder (F10-F19) [17]. |
| Intervention/exposure | Outpatient services consisting of either: (i) mental health specialist care co-located in alcohol and other drug treatment service settings; (ii) alcohol and other drug specialist care co-located in mental healthcare service settings; or (iii) dedicated dual diagnosis services providing care from both mental healthcare and alcohol and other drug treatment specialists in the same service location. |
| Comparison | Although we are not conducting a quantitative synthesis, where available our primary comparator for the qualitative synthesis of this review will be adults (≥18 years) with a dual diagnosis of mental illness and substance use disorder who:  1) Received no mental health or alcohol and other drug treatment;  2) Received either mental health or alcohol and other drug treatment alone; or  3) Received parallel treatment (i.e., are referred between separate mental healthcare and alcohol and other drug treatment service locations) or receive non-physically co-located mental healthcare and alcohol treatment. |
| Outcome | We defined five *a priori* primary outcomes of interest:  1) Treatment engagement patterns (i.e., treatment retention);  2) Substance use patterns and harms (i.e., frequency/severity of substance use, abstinence from substance use, or relapse);  3) Physical and mental health outcomes (i.e., rates of morbidity or mortality);  4) Other health service use patterns (i.e., primary care or acute care); and  5) Health economic outcomes (i.e., total costs or cost-effectiveness). |

PICO, patient/population, intervention, comparison and outcomes.

## **Table S2. Search strategy used for identifying relevant articles from MEDLINE® (Ovid)**

| **Line number** | **Search** |
| --- | --- |
| 1 | exp “Diagnosis, Dual (Psychiatry)”/ or exp comorbidity/ |
| 2 | ((dual or dually or concurrent or co-occur* or co-morbid* or multi-morbid* or cooccur* or comorbid* or multimorbid*) adj4 (diagnos* or disorder*)).mp. |
| 3 | (“chemically abusing mentally ill” or “chemically addicted and mentally ill” or CAMI or “co-occurring mental illness* and substance disorder*” or “cooccurring mental illness* and substance disorder*” or “dual substance and mental illness*” or “mentally ill chemical abuser” or “mentally ill chemically affected” or “mentally ill chemically addicted” or MICA or MICAA or “mentally ill substance abuser” or MISA or “mentally ill substance using” or MISU or “substance abusing mentally ill” or SAMI or “mentally ill chemically dependent” or MICD).mp. |
| 4 | exp anxiety disorders/ or exp “bipolar and related disorders”/ or exp “disruptive, impulse control, and conduct disorders”/ or exp dissociative disorders/ or exp elimination disorders/ or exp “feeding and eating disorders”/ or exp mood disorders/ or exp motor disorders/ or exp neurocognitive disorders/ or exp neurodevelopmental disorders/ or exp neurotic disorders/ or exp paraphilic disorders/ or exp personality disorders/ or exp “schizophrenia spectrum and other psychotic disorders”/ or exp sexual dysfunctions, psychological/ or exp sleep wake disorders/ or exp somatoform disorders/ or exp “trauma and stressor related disorders”/ |
| 5 | ((mental* or psych* or delusion* or personality or affective or anxious* or depressi* or conduct or defian* or clinical or emotional or generalized or generalised or mood or separation or panic or nervous or bipolar* or disruptive or impulse or conduct or dissociative or identity or elimination or encopresis or enuresis or feeding or eating or avoidant or restrictive or dysthymic or cyclothymic or neurocognitive or auditory or perceptual or consciousness or “attention deficit hyperactiv*” or manipulative or antisocial or borderline or compulsiv* or dependent or “passive aggressive” or capgras or sexual or gender or somatoform or “body integrity” or conversion or factitious or munchausen or “sleep wake” or trauma* or stress* or adjustment or combat) adj4 (diagnos* or disorder* or illness* or syndrome* or behaviour* or behavior* or delusion*)).mp. |
| 6 | (anxiety or agoraphobi* or “neurocirculatory asthenia” or neurotic or neuros* or psychoneuros* or “obsessive compulsi*” or hoarding or phobi* or bipolar or delusion* or “fire setting” or firesetting or gambling or trichotillomani* or “anorexia nervosa” or “binge eating” or “bulimia nervosa” or diabulimia or “food addiction” or “night eating” or Pica or amnesia or delirium or ADHD or tic* or tourette* or paraphili* or exhibitionis* or fetis* or masochis* or pedophili* or paedophili* or sadis* or transvestis* or voyeur* or BPD or histrionic or hysteria or parasitosis or alcoholic or schizo* or paranoi* or dyspareunia or “gender dysphori*” or “body dysmorphi*” or hypochondriasis or neurasthenia or dyssomni* or parasomni* or “psychological trauma” or “post-traumatic stress” or PTSD or “substance-induced psycho*” or “drug-induced psycho*”).mp. |
| 7 | *Substance-Related Disorders/ |
| 8 | exp alcohol-related disorders/ or exp amphetamine-related disorders/ or exp cocaine-related disorders/ or exp inhalant abuse/ or exp marijuana abuse/ or exp narcotic-related disorders/ or exp phencyclidine abuse/ or exp substance abuse, intravenous/ or exp substance abuse, oral/ or exp “tobacco use disorder”/ |
| 9 | ((drug* or alcohol* or drink* or alcohol* or chemical* or stimulant* or substance* or “substance-related” or oral or inhalant* or intravenous or inject* or “poly-drug*” or polydrug* or “poly-substance*” or polysubstance or poly or psychotropic or opiate* or opioid* or cannabis or marijuana or marihuana or satvex or benzodiazepine* or dipsomani or legal or illicit or “non-prescribed” or nonprescribed or cocain* or codrenine or “ecgonine methyl ester benzoate” or erythroxylin or locosthetic or neurocaine or sterilocain or narcotic* or heroin or diacetylmorphin* or diagesil or diamorf or diamorphin* or solvent* or psychotropic or stimulant* or psychostimulant* or inhalant* or narcotic* or morphine or phencyclidine or valium or “crystal meth” or “crystal methamphetamine*” or “benzoylmethyl ecgonine” or “pain medication” or desoxyn or dexamfetamin* or dexedrine or dextroamphetamine* or amphetamine* or methamphetamine* or oral or tobacco or nicotine) adj4 (addict* or abus* or misus* or use* or using or dependenc* or problem* or habit* or disorder* or behaviour* or behavior*)).mp. |
| 10 | Alcoholic*.mp. |
| 11 | 1 or 2 or 3 |
| 12 | 4 or 5 or 6 |
| 13 | 7 or 8 or 9 or 10 |
| 14 | 12 and 13 |
| 15 | 11 or 14 |
| 16 | ((integrat* or co-locat* or colocat* or embed*) adj4 (care or intervention* or regimen* or treatment* or therap* or servic* or program* or rehabil*)).mp. |
| 17 | 15 and 16 |
| 18 | limit 17 to “all adult (19 plus years)” |

## **Table S3. Search strategy used for identifying relevant articles from EMBASE**

| **Line number** | **Search** |
| --- | --- |
| 1 | exp comorbidity/ |
| 2 | ((dual or dually or concurrent or co-occur* or co-morbid* or multi-morbid* or cooccur* or comorbid* or multimorbid*) adj4 (diagnos* or disorder*)).mp. |
| 3 | (“chemically abusing mentally ill” or “chemically addicted and mentally ill” or CAMI or “co-occurring mental illness* and substance disorder*” or “cooccurring mental illness* and substance disorder*” or “dual substance and mental illness*” or “mentally ill chemical abuser” or “mentally ill chemically affected” or “mentally ill chemically addicted” or MICA or MICAA or “mentally ill substance abuser” or MISA or “mentally ill substance using” or MISU or “substance abusing mentally ill” or SAMI or “mentally ill chemically dependent” or MICD).mp. |
| 4 | exp adjustment disorder/ or exp alexithymia/ or exp anxiety disorder/ or exp autism/ or exp behavior disorder/ or exp complicated grief/ or exp confusion/ or exp delirium/ or exp dissociative disorder/ or exp emotional disorder/ or exp experimental mental disease/ or exp hikikomori/ or exp learning disorder/ or exp memory disorder/ or exp mental deficiency/ or exp mental infantilism/ or exp mental instability/ or exp mental overstimulation/ or exp mood disorder/ or exp neurosis/ or exp organic brain syndrome/ or exp organic psychosyndrome/ or exp personality disorder/ or exp psychosexual disorder/ or exp psychosis/ or exp psychosomatic disorder/ or exp psychotrauma/ or exp schizophrenia spectrum disorder/ or exp stupor/ or exp thought disorder/ |
| 5 | ((mental* or psych* or delusion* or personality or affective or anxious* or depressi* or conduct or defian* or clinical or emotional or generalized or generalised or mood or separation or panic or nervous or bipolar* or disruptive or impulse or conduct or dissociative or identity or elimination or encopresis or enuresis or feeding or eating or avoidant or restrictive or dysthymic or cyclothymic or neurocognitive or auditory or perceptual or consciousness or “attention deficit hyperactiv*” or manipulative or antisocial or borderline or compulsiv* or dependent or “passive aggressive” or capgras or sexual or gender or somatoform or “body integrity” or conversion or factitious or munchausen or “sleep wake” or trauma* or stress* or adjustment or combat) adj4 (diagnos* or disorder* or illness* or syndrome* or behavior* or behaviour* or delusion*)).mp. |
| 6 | (anxiety or agoraphobi* or “neurocirculatory asthenia” or neurotic or neuros* or psychoneuros* or “obsessive compulsi*” or hoarding or phobi* or bipolar or delusion* or “fire setting” or firesetting or gambling or trichotillomani* or “anorexia nervosa” or “binge eating” or “bulimia nervosa” or diabulimia or “food addiction” or “night eating” or Pica or amnesia or delirium or ADHD or tic* or tourette* or paraphili* or exhibitionis* or fetis* or masochis* or pedophili* or paedophili* or sadis* or transvestis* or voyeur* or BPD or histrionic or hysteria or parasitosis or alcoholic or schizo* or paranoi* or dyspareunia or “gender dysphori*” or “body dysmorphi*” or hypochondriasis or neurasthenia or dyssomni* or parasomni* or “psychological trauma” or “post-traumatic stress” or PTSD or “substance-induced psycho*” or “drug-induced psycho*”).mp. |
| 7 | exp drug dependence/ |
| 8 | ((drug* or alcohol* or drink* or alcohol* or chemical* or stimulant* or substance* or “substance-related” or oral or inhalant* or intravenous or inject* or “poly-drug*” or polydrug* or “poly-substance*” or polysubstance or poly or psychotropic or opiate* or opioid* or cannabis or marijuana or marihuana or satvex or benzodiazepine* or dipsomani or legal or illicit or “non-prescribed” or nonprescribed or cocain* or codrenine or “ecgonine methyl ester benzoate” or erythroxylin or locosthetic or neurocaine or sterilocain or narcotic* or heroin or diacetylmorphin* or diagesil or diamorf or diamorphin* or solvent* or psychotropic or stimulant* or psychostimulant* or inhalant* or narcotic* or morphine or phencyclidine or valium or “crystal meth” or “crystal methamphetamine*” or “benzoylmethyl ecgonine” or “pain medication” or desoxyn or dexamfetamin* or dexedrine or dextroamphetamine* or amphetamine* or methamphetamine* or oral or tobacco or nicotine) adj4 (addict* or abus* or misus* or use* or using or dependenc* or problem* or habit* or disorder* or behavior* or behaviour*)).mp. |
| 9 | Alcoholic*.mp. |
| 10 | 1 or 2 or 3 |
| 11 | 4 or 5 or 6 |
| 12 | 7 or 8 or 9 |
| 13 | 11 and 12 |
| 14 | 10 or 13 |
| 15 | ((integrat* or co-locat* or colocat* or embed*) adj4 (care or intervention* or regimen* or treatment* or therap* or servic* or program* or rehabil*)).mp |
| 16 | 14 and 15 |
| 17 | limit 16 to adult <18 to 64 years> |
| 18 | limit 17 to aged <65+ years> |
| 19 | 17 or 18 |

## **Table S4. Search strategy used for identifying relevant articles from PsycINFO**

| **Line number** | **Search** |
| --- | --- |
| 1 | exp Dual Diagnosis/ or exp comorbidity/ |
| 2 | ((dual or dually or concurrent or co-occur* or co-morbid* or multi-morbid* or cooccur* or comorbid* or multimorbid*) adj4 (diagnos* or disorder*)).mp. |
| 3 | (“chemically abusing mentally ill” or “chemically addicted and mentally ill” or CAMI or “co-occurring mental illness* and substance disorder*” or “cooccurring mental illness* and substance disorder*” or “dual substance and mental illness*” or “mentally ill chemical abuser” or “mentally ill chemically affected” or “mentally ill chemically addicted” or MICA or MICAA or “mentally ill substance abuser” or MISA or “mentally ill substance using” or MISU or “substance abusing mentally ill” or SAMI or “mentally ill chemically dependent” or MICD).mp. |
| 4 | exp mental disorders/ |
| 5 | ((mental* or psych* or delusion* or personality or affective or anxious* or depressi* or conduct or defian* or clinical or emotional or generalized or generalised or mood or separation or panic or nervous or bipolar* or disruptive or impulse or conduct or dissociative or identity or elimination or encopresis or enuresis or feeding or eating or avoidant or restrictive or dysthymic or cyclothymic or neurocognitive or auditory or perceptual or consciousness or “attention deficit hyperactiv*” or manipulative or antisocial or borderline or compulsiv* or dependent or “passive aggressive” or capgras or sexual or gender or somatoform or “body integrity” or conversion or factitious or munchausen or “sleep wake” or trauma* or stress* or adjustment or combat) adj4 (diagnos* or disorder* or illness* or syndrome* or behavior* or behaviour* or delusion*)).mp. |
| 6 | (anxiety or agoraphobi* or “neurocirculatory asthenia” or neurotic or neuros* or psychoneuros* or “obsessive compulsi*” or hoarding or phobi* or bipolar or delusion* or “fire setting” or firesetting or gambling or trichotillomani* or “anorexia nervosa” or “binge eating” or “bulimia nervosa” or diabulimia or “food addiction” or “night eating” or Pica or amnesia or delirium or ADHD or tic* or tourette* or paraphili* or exhibitionis* or fetis* or masochis* or pedophili* or paedophili* or sadis* or transvestis* or voyeur* or BPD or histrionic or hysteria or parasitosis or alcoholic or schizo* or paranoi* or dyspareunia or “gender dysphori*” or “body dysmorphi*” or hypochondriasis or neurasthenia or dyssomni* or parasomni* or “psychological trauma” or “post-traumatic stress” or PTSD or “substance-induced psycho*” or “drug-induced psycho*”).mp. |
| 7 | exp drug abuse/ |
| 8 | ((drug* or alcohol* or drink* or alcohol* or chemical* or stimulant* or substance* or “substance-related” or oral or inhalant* or intravenous or inject* or “poly-drug*” or polydrug* or “poly-substance*” or polysubstance or poly or psychotropic or opiate* or opioid* or cannabis or marijuana or marihuana or satvex or benzodiazepine* or dipsomani or legal or illicit or “non-prescribed” or nonprescribed or cocain* or codrenine or “ecgonine methyl ester benzoate” or erythroxylin or locosthetic or neurocaine or sterilocain or narcotic* or heroin or diacetylmorphin* or diagesil or diamorf or diamorphin* or solvent* or psychotropic or stimulant* or psychostimulant* or inhalant* or narcotic* or morphine or phencyclidine or valium or “crystal meth” or “crystal methamphetamine*” or “benzoylmethyl ecgonine” or “pain medication” or desoxyn or dexamfetamin* or dexedrine or dextroamphetamine* or amphetamine* or methamphetamine* or oral or tobacco or nicotine) adj4 (addict* or abus* or misus* or use* or using or dependenc* or problem* or habit* or disorder* or behavior* or behaviour*)).mp. |
| 9 | Alcoholic*.mp. |
| 10 | 1 or 2 or 3 |
| 11 | 4 or 5 or 6 |
| 12 | 7 or 8 or 9 |
| 13 | 11 and 12 |
| 14 | 10 or 13 |
| 15 | ((integrat* or co-locat* or colocat* or embed*) adj4 (care or intervention* or regimen* or treatment* or therap* or servic* or program* or rehabil*)).mp. |
| 16 | 14 and 15 |
| 17 | limit 16 to adulthood <18+ years> |

## **Table S5. Search strategy used for identifying relevant articles from CINAHL**

| **Line number** | **Search** |
| --- | --- |
| S1 | MH (“comorbidity”) |
| S2 | TI (dual or dually or concurrent or co-occur* or co-morbid* or multi-morbid* or cooccur* or comorbid* or multimorbid*) n4 (diagnos* or disorder*) |
| S3 | AB (dual or dually or concurrent or co-occur* or co-morbid* or multi-morbid* or cooccur* or comorbid* or multimorbid*) n4 (diagnos* or disorder*) |
| S4 | TI (“chemically abusing mentally ill” or “chemically addicted and mentally ill” or CAMI or “co-occurring mental illness* and substance disorder*” or “cooccurring mental illness* and substance disorder*” or “dual substance and mental illness*” or “mentally ill chemical abuser” or “mentally ill chemically affected” or “mentally ill chemically addicted” or MICA or MICAA or “mentally ill substance abuser” or MISA or “mentally ill substance using” or MISU or “substance abusing mentally ill” or SAMI or “mentally ill chemically dependent” or MICD) |
| S5 | AB (“chemically abusing mentally ill” or “chemically addicted and mentally ill” or CAMI or “co-occurring mental illness* and substance disorder*” or “cooccurring mental illness* and substance disorder*” or “dual substance and mental illness*” or “mentally ill chemical abuser” or “mentally ill chemically affected” or “mentally ill chemically addicted” or MICA or MICAA or “mentally ill substance abuser” or MISA or “mentally ill substance using” or MISU or “substance abusing mentally ill” or SAMI or “mentally ill chemically dependent” or MICD) |
| S6 | (MH “Mental Disorders”) OR (MH “Adjustment Disorders+”) OR (MH “Intellectual Disability+”) OR (MH “Mental Disorders Diagnosed in Childhood+”) OR (MH “Neurotic Disorders+”) OR (MH “Olfactory Reference Syndrome”) OR (MM “Organic Mental Disorders”) OR (MH “Organic Mental Disorders, Psychotic+”) OR (MH “Personality Disorders+”) OR (MH “Pregnancy Complications, Psychiatric+”) OR (MH “Psychophysiologic Disorders+”) OR (MH “Psychotic Disorders+”) OR (MH “Sexual and Gender Disorders+”) |
| S7 | TI (mental* or psych* or delusion* or personality or affective or anxious* or depressi* or conduct or defian* or clinical or emotional or generalized or generalised or mood or separation or panic or nervous or bipolar* or disruptive or impulse or conduct or dissociative or identity or elimination or encopresis or enuresis or feeding or eating or avoidant or restrictive or dysthymic or cyclothymic or neurocognitive or auditory or perceptual or consciousness or “attention deficit hyperactiv*” or manipulative or antisocial or borderline or compulsiv* or dependent or “passive aggressive” or capgras or sexual or gender or somatoform or “body integrity” or conversion or factitious or munchausen or “sleep wake” or trauma* or stress* or adjustment or combat) n4 (diagnos* or disorder* or illness* or syndrome* or behaviour* or behavior* or delusion*) |
| S8 | AB (mental* or psych* or delusion* or personality or affective or anxious* or depressi* or conduct or defian* or clinical or emotional or generalized or generalised or mood or separation or panic or nervous or bipolar* or disruptive or impulse or conduct or dissociative or identity or elimination or encopresis or enuresis or feeding or eating or avoidant or restrictive or dysthymic or cyclothymic or neurocognitive or auditory or perceptual or consciousness or “attention deficit hyperactiv*” or manipulative or antisocial or borderline or compulsiv* or dependent or “passive aggressive” or capgras or sexual or gender or somatoform or “body integrity” or conversion or factitious or munchausen or “sleep wake” or trauma* or stress* or adjustment or combat) n4 (diagnos* or disorder* or illness* or syndrome* or behaviour* or behaviour* or delusion*) |
| S9 | TI (anxiety or agoraphobi* or “neurocirculatory asthenia” or neurotic or neuros* or psychoneuros* or “obsessive compulsi*” or hoarding or phobi* or bipolar or delusion* or “fire setting” or firesetting or gambling or trichotillomani* or “anorexia nervosa” or “binge eating” or “bulimia nervosa” or diabulimia or “food addiction” or “night eating” or Pica or amnesia or delirium or ADHD or tic* or tourette* or paraphili* or exhibitionis* or fetis* or masochis* or pedophili* or paedophili* or sadis* or transvestis* or voyeur* or BPD or histrionic or hysteria or parasitosis or alcoholic or schizo* or paranoi* or dyspareunia or “gender dysphori*” or “body dysmorphi*” or hypochondriasis or neurasthenia or dyssomni* or parasomni* or “psychological trauma” or “post-traumatic stress” or PTSD or “substance-induced psycho*” or “drug-induced psycho*”) |
| S10 | AB (anxiety or agoraphobi* or “neurocirculatory asthenia” or neurotic or neuros* or psychoneuros* or “obsessive compulsi*” or hoarding or phobi* or bipolar or delusion* or “fire setting” or firesetting or gambling or trichotillomani* or “anorexia nervosa” or “binge eating” or “bulimia nervosa” or diabulimia or “food addiction” or “night eating” or Pica or amnesia or delirium or ADHD or tic* or tourette* or paraphili* or exhibitionis* or fetis* or masochis* or pedophili* or paedophili* or sadis* or transvestis* or voyeur* or BPD or histrionic or hysteria or parasitosis or alcoholic or schizo* or paranoi* or dyspareunia or “gender dysphori*” or “body dysmorphi*” or hypochondriasis or neurasthenia or dyssomni* or parasomni* or “psychological trauma” or “post-traumatic stress” or PTSD or “substance-induced psycho*” or “drug-induced psycho*”) |
| S11 | (MH “Substance Use Disorders+”) |
| S12 | TI (drug* or alcohol* or drink* or alcohol* or chemical* or stimulant* or substance* or “substance-related” or oral or inhalant* or intravenous or inject* or “poly-drug*” or polydrug* or “poly-substance*” or polysubstance or poly or psychotropic or opiate* or opioid* or cannabis or marijuana or marihuana or satvex or benzodiazepine* or dipsomani or legal or illicit or “non-prescribed” or nonprescribed or cocain* or codrenine or “ecgonine methyl ester benzoate” or erythroxylin or locosthetic or neurocaine or sterilocain or narcotic* or heroin or diacetylmorphin* or diagesil or diamorf or diamorphin* or solvent* or psychotropic or stimulant* or psychostimulant* or inhalant* or narcotic* or morphine or phencyclidine or valium or “crystal meth” or “crystal methamphetamine*” or “benzoylmethyl ecgonine” or “pain medication” or desoxyn or dexamfetamin* or dexedrine or dextroamphetamine* or amphetamine* or methamphetamine* or oral or tobacco or nicotine) n4 (addict* or abus* or misus* or use* or using or dependenc* or problem* or habit* or disorder* or behaviour* or behaviour*) |
| S13 | AB (drug* or alcohol* or drink* or alcohol* or chemical* or stimulant* or substance* or “substance-related” or oral or inhalant* or intravenous or inject* or “poly-drug*” or polydrug* or “poly-substance*” or polysubstance or poly or psychotropic or opiate* or opioid* or cannabis or marijuana or marihuana or satvex or benzodiazepine* or dipsomani or legal or illicit or “non-prescribed” or nonprescribed or cocain* or codrenine or “ecgonine methyl ester benzoate” or erythroxylin or locosthetic or neurocaine or sterilocain or narcotic* or heroin or diacetylmorphin* or diagesil or diamorf or diamorphin* or solvent* or psychotropic or stimulant* or psychostimulant* or inhalant* or narcotic* or morphine or phencyclidine or valium or “crystal meth” or “crystal methamphetamine*” or “benzoylmethyl ecgonine” or “pain medication” or desoxyn or dexamfetamin* or dexedrine or dextroamphetamine* or amphetamine* or methamphetamine* or oral or tobacco or nicotine) n4 (addict* or abus* or misus* or use* or using or dependenc* or problem* or habit* or disorder* or behaviour* or behaviour*) |
| S14 | TI (Alcoholic*) or AB (Alcoholic*) |
| S15 | S1 or S2 or S3 or S4 or S5 |
| S16 | S6 or S7 or S8 or S9 or S10 |
| S17 | S11 or S12 or S13 or S14 |
| S18 | S16 and S17 |
| S19 | S15 or S18 |
| S20 | TI (integrat* or co-locat* or colocat* or embed*) n4 (care or intervention* or regimen* or treatment* or therap* or servic* or program* or rehabil*) |
| S21 | AB (integrat* or co-locat* or colocat* or embed*) n4 (care or intervention* or regimen* or treatment* or therap* or servic* or program* or rehabil*) |
| S22 | S20 or S21 |
| S23 | S19 and S22 |
| S24 | Applied “all adults” filter |

## **Table S6. Search strategy used for identifying relevant articles from Web of Science**

| **Line number** | **Search** |
| --- | --- |
| 1 | TS=((dual or dually or concurrent or co-occur* or co-morbid* or multi-morbid* or cooccur* or comorbid* or multimorbid*) NEAR/4 (diagnos* or disorder*) ) |
| 2 | TS=(“chemically abusing mentally ill” or “chemically addicted and mentally ill” or CAMI or “co-occurring mental illness* and substance disorder*” or “cooccurring mental illness* and substance disorder*” or “dual substance and mental illness*” or “mentally ill chemical abuser” or “mentally ill chemically affected” or “mentally ill chemically addicted” or MICA or MICAA or “mentally ill substance abuser” or MISA or “mentally ill substance using” or MISU or “substance abusing mentally ill” or SAMI or “mentally ill chemically dependent” or MICD) |
| 3 | TS=((mental* or psych* or delusion* or personality or affective or anxious* or depressi* or conduct or defian* or clinical or emotional or generalized or generalised or mood or separation or panic or nervous or bipolar* or disruptive or impulse or conduct or dissociative or identity or elimination or encopresis or enuresis or feeding or eating or avoidant or restrictive or dysthymic or cyclothymic or neurocognitive or auditory or perceptual or consciousness or “attention deficit hyperactiv*” or manipulative or antisocial or borderline or compulsiv* or dependent or “passive aggressive” or capgras or sexual or gender or somatoform or “body integrity” or conversion or factitious or munchausen or “sleep wake” or trauma* or stress* or adjustment or combat) NEAR/4 (diagnos* or disorder* or illness* or syndrome* or behavior* or behaviour* or delusion*) ) |
| 4 | TS=(anxiety or agoraphobi* or “neurocirculatory asthenia” or neurotic or neuros* or psychoneuros* or “obsessive compulsi*” or hoarding or phobi* or bipolar or delusion* or “fire setting” or firesetting or gambling or trichotillomani* or “anorexia nervosa” or “binge eating” or “bulimia nervosa” or diabulimia or “food addiction” or “night eating” or Pica or amnesia or delirium or ADHD or tic* or tourette* or paraphili* or exhibitionis* or fetis* or masochis* or pedophili* or paedophili* or sadis* or transvestis* or voyeur* or BPD or histrionic or hysteria or parasitosis or alcoholic or schizo* or paranoi* or dyspareunia or “gender dysphori*” or “body dysmorphi*” or hypochondriasis or neurasthenia or dyssomni* or parasomni* or “psychological trauma” or “post-traumatic stress” or PTSD or “substance-induced psycho*” or “drug-induced psycho*”) |
| 5 | TS=((drug* or alcohol* or drink* or alcohol* or chemical* or stimulant* or substance* or “substance-related” or oral or inhalant* or intravenous or inject* or “poly-drug*” or polydrug* or “poly-substance*” or polysubstance or poly or psychotropic or opiate* or opioid* or cannabis or marijuana or marihuana or satvex or benzodiazepine* or dipsomani or legal or illicit or “non-prescribed” or nonprescribed or cocain* or codrenine or “ecgonine methyl ester benzoate” or erythroxylin or locosthetic or neurocaine or sterilocain or narcotic* or heroin or diacetylmorphin* or diagesil or diamorf or diamorphin* or solvent* or psychotropic or stimulant* or psychostimulant* or inhalant* or narcotic* or morphine or phencyclidine or valium or “crystal meth” or “crystal methamphetamine*” or “benzoylmethyl ecgonine” or “pain medication” or desoxyn or dexamfetamin* or dexedrine or dextroamphetamine* or amphetamine* or methamphetamine* or oral or tobacco or nicotine) NEAR/4 (addict* or abus* or misus* or use* or using or dependenc* or problem* or habit* or disorder* or behavior* or behaviour*) ) |
| 6 | TS=(Alcoholic*) |
| 7 | #1 or #2 |
| 8 | #3 or #4 |
| 9 | #5 or #6 |
| 10 | #8 and #9 |
| 11 | #7 or #10 |
| 12 | TS=(( integrat* or co-locat* or colocat* or embed*) NEAR/4 (care or intervention* or regimen* or treatment* or therap* or servic* or program* or rehabil*) ) |
| 13 | #11 and #12 |

**Table S7. Study inclusion and exclusion criteria**

| **Inclusion criteria** |
| --- |
| 1) Peer-reviewed literature. |
| 2) Reported in English. |
| 3) Studies with human subjects. |
| 4) Studies with adults (≥18 years) seeking outpatient treatment for a dual diagnosis of mental illness and substance use disorder, both with and without evidence of a formal diagnosis. |
| 5) Observational studies or randomised controlled trials. |
| **Exclusion criteria** |
| 1) Wrong exposure - no evidence of mental healthcare and alcohol and other drug treatment service co-location. |
| 2) Wrong outcome - no reporting of a) treatment engagement patterns (i.e., treatment retention); b) substance use patterns or harms (i.e., substance use abstinence or relapse); c) health outcomes (i.e., morbidity or mortality); d) other service use patterns (i.e., primary care or acute care); or e) health economic outcomes (i.e., costs or cost-effectiveness). |
| 3) Studies of individuals (i.e., case studies and case reports), observational studies where there is no person-level analysis (i.e., ecological studies), and previous systematic reviews. |
| 4) Studies which exclusively sampled from inpatient services, did not disaggregate outcomes between inpatient and outpatient samples, or which exclusively examined specialist treatment embedded in primary care settings. |
| 5) Studies exclusively conducted on children or adolescents (<18 years). |
| 6) Studies in which no quantitative data were reported. |

**Table S8. Summary of outcomes reported in the included studies**

| **Study** | **Treatment engagement patterns** | **Substance use patterns and harms** | **Health outcomes** | **Health service use outcomes** | **Health economic outcomes** |
| --- | --- | --- | --- | --- | --- |
| Bartels et al. 2004 | Individuals in the co-located model had higher odds of treatment engagement than among those in the control group (OR 2.6; 95% CI 2.1-3.1). After adjusting for symptom severity, the adjusted OR for treatment engagement comparing those in co-located care to standard care was 3.19 (p<0.001, no 95% CI reported). Compared with the standard care group, individuals who received co-located care were more likely to return for subsequent treatment visits (53.6% vs. 30.4%; p<0.001). Those in the co-located treatment condition with a dual diagnosis of depression/anxiety and alcohol use disorder had increased odds of treatment engagement compared to their counterparts who received standard care (OR 3.7; 95% CI 1.8-7.6) | Not reported | Not reported | Not reported | Not reported |
| Bhalla et al. 2020 | Veterans receiving treatment at the integrated care clinic attended more appointments across all appointment types – psychiatry, substance abuse, medical/surgical, primary care and specialty – than those receiving standard care. They also attended more outpatient appointments overall (20.2 (SD±23.8) vs. 59.9 (SD±63.8) than the standard care group. | Not reported | Not reported | After adjusting for differences in clinical severity, veterans receiving integrated care were more likely to receive inpatient mental healthcare (OR 1.79, p<0.001) and to attend general psychiatry appointments (OR 1.01, p<0.001) and substance abuse outpatient visits (OR 1.01, p=0.001) than those receiving standard care. | Not reported |
| Bond et al. 1991 | Treatment engagement after 12 months was 83% (19/23) for group therapy participants, 81% (25/31) for ACT participants and 47% (20/43) of control participants. After 18 months, treatment engagement rates for both the group therapy (p<0.001) and ACT (p<0.05) were greater than controls. Through the first 12 months of treatment, group therapy participants had an average of 255 treatment contact hours, compared with 56 hours for ACT participants and 16 hours for controls. | There was a statistically significant within-group reduction for group therapy participants at 6-months (t(17)=2.3, p<0.05). Additionally, there was a significant treatment by time interaction (F(6,147)=2.3, p<0.05) for reduction in cannabis use for reference group participants compared to controls. | There were significant improvements in the Quality of Life as measured by the Life Satisfaction Checklist for reference group participants at 6 months (t(17)=2.8, p<0.05) and at 12 months (t(18)=2.8, p<0.05) | Multivariate ANOVA showed a significant effect for treatment (F(2,79)=3.4, p<0.05) and an effect for time (F(3,237)=2.3, p<0.08), but no treatment by time interaction. There were between group differences at 6 months in univariate ANOVA comparisons (F(2,89)=3.3, p<0/05) and also at 12 months (F(2,87)=3.3, p<0.05). Post hoc tests showed that participants in the reference group condition had significantly fewer hospitalisations than participants in either the ACT or control groups at both 6 and 12 months follow up. ACT participants demonstrated a reduction in hospital days at 6 months (t(27)=3.0, p<0.01) and 18 months (t(24)=2.5, p<0.05) Both control group and reference group participants showed a reduction in hospitalisation days at 6 months | Not reported |
| Bouchery et al. 2018 | Not reported | Not reported | Not reported | Those receiving co-located care had 0.02 (95% CI 0.01-0.02) fewer hospitalisations (p<0.01), 0.03 (95% CI 0.01-0.05) fewer ED visits (p=0.01), and 0.13 (95% CI 0.03-0.23) fewer office visits (p=0.04) per month of enrolment attributed to the co-located care model compared to the standard care group. Over the observation period (2.5 years) this translated to one less hospitalisation for every two clients served, five fewer ED visits for every six clients served and four fewer office visits for every one client served | Over the observation period (2.5 years) Medicare expenditures decreased on average by USD$266 (95% CI $69-$463) per enrolled participant per month for the co-located care group when compared to the standard care group (p<0.01) |
| Brooner et al. 2013 | Participants in the co-located care group versus the standard care condition were more likely to begin psychiatric medications (90% vs. 82%; p=0.032). SSRI antidepressants were prescribed most often, followed by heterocyclic antidepressants and atypical antipsychotics. Participants receiving co-located psychiatric and substance misuse care were more likely to complete the initial psychiatric intake (OR 4.0, 95% CI 3.1–5.1) and progressed to intake from initial assessment in fewer days (median = 5.0 compared to median = 31.0) than participants receiving off-site psychiatric care. Participants receiving co-located psychiatric and substance misuse care also had more mean days of psychiatric treatment (196 vs. 102; p<0.001) and were more likely to complete the 1-year episode when compared to the standard care group (OR 3.6, 95% CI 2.0–6.3) | Not reported | Co-located care participants had lower mean psychological distress at follow-up as measured by the Hopkins Severity Index (SCL-90-R GSI scores on-site: mean = 43; SE = 0.6 vs. off-site: mean = 46; SE = 0.6; p=0.006). Co-located care participants attained greater reductions in GSI change scores (mean reduction = 4.2; SE = 0.6 vs. standard care mean reduction = 1.7; SE = 0.6; p=0.003) | Not reported | Not reported |
| Clark et al. 1998 | Not reported | Not reported | Not reported | Not reported | ACT participants showed a trend towards lower costs after 3 years in mental health inpatient treatment (USD$27,604 compared with USD$34,006) and general health inpatient treatment (USD$645 compared with USD$1,345). During the first year of observation, SCM produced better outcomes per USD$10,000 invested than ACT. However, during the last year of observation ACT produced substantially better outcomes per USD$10,000 investment than SCM |
| Clausen et al. 2020 | Not reported | 7% (6/84) of participants with problematic substance use at enrolment no longer met criteria for problematic substance use at follow up. However, 7% (4/58) who did not have problematic substance use at enrolment met criteria for problematic substance use after 2 years of ACT | The reduction of BPRS agitation/mania symptoms was significantly greater among participants with problematic substance use (baseline=2.42, follow-up=2.07) than among participants without problematic substance use (baseline=1.78, follow-up=1.78, p=0.010). Participants with co-occurring disorders had demonstrated reduced mean symptom severity scores on the BPRS overall (baseline=2.60, follow-up=2.38, p=0.020). They also showed improvements in the mean score on the negative symptoms (baseline=2.43, follow-up=2.07, p=0.019), agitation/mania (baseline=2.42, follow-up=2.07, p=0.001) and anxiety/depression (baseline=2.77, follow-up=2.43, p=0.005) scales. There was no significant change in mean positive symptoms scale score (p=0.784). There were significant improvements in level of functioning as measured by the Global Assessment of Functioning for participants with co-occurring disorders (baseline=38.9, follow-up=42.6, p=0.016) and participants with mental illness alone (baseline=40.8, follow-up=44.7, p=0.036). There were no significant improvements in quality of life as measured by the Manchester Short Assessment of Quality of Life in for participants with co-occurring disorders (p=0.541) or mental illness alone (p=0.203) | Not reported. | Not reported. |
| Cooper et al. 2010 | Not reported | After 6 months the rate of relapse to substance use was 35% and this reduced to 28% at 12 months. 56% of participants were abstinent from illicit substances at both 6 and 12 months. Alcohol use significantly decreased across time (F (1.5, 498) = 57.3, p<0.01) and use of illicit drugs also significantly reduced over time (F (1.7, 553) = 94.3, p<0.01) with both cocaine use (F (1.6, 539) = 50.3, p<0.01) and cannabis use (F (1.6, 532) = 30.3, p<0.01) decreasing significantly. There was also a significant reduction in drug-related harm as measured by a 4-point Likert scale assessing drug related stress (Friedman's test χ2(2) = 117, p=0.0001), drug-use activities (χ2(2) = 156, p=0.0001) and drug-related emotional problems (χ2(2) = 141, p=0.0001) | Severity of psychiatric symptoms as measured by the Brief Symptom Inventory was reduced significantly after 6 months, and the reduction was maintained at 1-year follow-up (F (1.9, 299.2) = 43.7, p=0.0001) for participants receiving co-located care | Emergency department attendances for mental health services decreased significantly across all follow-up points (F(1.9, 279) = 3.1, p=0.05). Significant decreases in all service utilisation were found for inpatient mental health (F(1.5, 219) = 8.1, p=0.001) and in outpatient mental health service use (F (1.7, 267) = 19.1, p=0.0001) across all follow-up time points | Not reported |
| Drake et al. 1993 | Not reported | 11 out of 18 participants (61%) were in remission (no evidence of alcohol use disorder for at least 6 months) at follow up.The mean length of remission was 26.5 months (SD = 13.5) | Not reported | Not reported | Not reported |
| Drake et al. 1998 | Not reported | There was no significant difference in the proportion of patients with alcohol use disorder who achieved abstinence during the study period in the co-located treatment model (43%) compared to standard care (50%; χ2 (1, n=142) = 0.65, not significant [NS]). Similarly, there was no difference in the proportion of patients who achieved abstinence in the co-located care model compared to standard care (43% vs. 35%; χ2 (1, n=84) = 0.6, NS) | Overall life satisfaction as measured by the Quality-of-Life Interview showed significant improvements over time (F (6,196) = 4.72, p<0.01); within-group time effects were significant for co-location patients and not for controls, indicating that co-location model of care accounted for the effects on this outcome | Both groups showed significant reductions in hospital admissions over 36 months, but no significant between group differences were observed (measure of effect and p-value not reported) | Not reported |
| Drake et al. 2004 | Not reported | Over the course of 3 years, 37 (73%) of the 51 patients achieved full abstinence (generalised estimating equation [GEE] p<0.01) (defined by DSM-III-R as at least 6 months without any signs of misuse or dependence). However, approximately one third of these (12 of 33; 36%) relapsed within 1 year (4 patients did not have sufficient follow-up time after achieving full abstinence to be followed for one year) | General life satisfaction, measured using the Quality of Life Interview improved significantly from 3.5 (SD = 1.4) at baseline to 4.7 (SD = 0.9) at the end of 3 years (p<0.01) | Participants decreased days hospitalised across the three follow-ups (p<0.01). Outpatient utilisation showed a curvilinear pattern (p<0.01), that is, participants decreased their inpatient hospital days as they became engaged in outpatient services and increased their outpatient service use | Not reported |
| Drake et al. 2016 | Not reported | Participants receiving co-located and integrated mental healthcare and AOD treatment had significantly increased remission of substance use disorder over follow-up (8% baseline and 61% at follow-up; p<0.01). Furthermore, abstinence during the past 6 months increased over follow-up (3% at baseline to 41% at year 7 follow-up; p<0.01) | Participants receiving co-located and integrated mental healthcare and AOD treatment had increased remission of psychiatric symptoms over follow-up (45% baseline vs. 70% at 7 years follow-up; p<0.01). Similarly, quality of life, as measured by general life satisfaction, increased significantly over follow-up (35% at baseline vs. 53% at 7 years follow-up; p<0.01) | The proportion of participants with a hospital admission in the past year significantly decreased over follow-up (60% at baseline, 27% at year 7 follow-up; p<0.01). The proportion of participants receiving individual outpatient services remained consistent over follow-up with no significant change observed (minimum: 62%, maximum 69%, p>0.05). A similar pattern was observed for outpatient group treatments (minimum: 34%, maximum: 56%, p>0.05) | Not reported |
| Fletcher et al. 2008 | Not reported | No difference was observed between the co-located treatment conditions and standard care on a 5-point substance misuse rating scale (ranging from 'no use' to 'severe use and related problems'; p>0.1) | There were no significant improvements in psychiatric symptoms for the co-located treatment conditions compared to standard care (p>0.1) | Not reported | Not reported |
| Frisman et al. 2009 | Not reported | Among participants with ASPD and substance use disorder, those assigned to the standard care group showed an increase in alcohol use (Alcohol Use Scale rating) over follow-up compared with those who were assigned to co-located care (beta= 0.03; SE = 0.01, p=0.0437) | Not reported | Not reported | Not reported |
| Granholm et al. 2003 | Not reported | Not reported | Not reported | Comparing the year before engagement in co-located care to the year after, a 60% reduction in the number of psychiatric hospitalisation days following treatment was found for the entire sample (1 year before mean=12.8±20.2 vs. 1 year after mean=5.1±9.1: F = 5.10, df = 1,41, p=0.029). The number of days receiving co-located treatment was not significantly correlated with a reduction in hospitalisation days (year before-year after difference) for psychiatric (r=-0.10) or SUD (r=0.20) hospitalisations (p>0.05) | Not reported |
| Holdcraft et al. 2002 | Not reported | The mean length of abstinence increased from 15 months at intake to 21 months at one-year of follow-up (t (14) = -3.0, p<0.05). In a sensitivity analysis restricted to participants with less than one year of abstinence before entering the program, a significant increase in abstinence was observed (mean of 5 years; p<0.05) | Participants’ rating of quality of life at a 1-year follow-up showed a non-significant numerical improvement (p>0.05) | The mean number of psychiatric hospitalisations reduced from 1.6 in the year prior to entering co-located treatment to 0.4 during the first year of co-located treatment (t (19) = 2.4, p<0.05) | Not reported |
| Judd et al. 2003 | Not reported | Over three years of follow-up, there was significant improvement on the Basis-32 Impulsive/Addictive scale (p<0.05). For participants who engaged in co-located treatment for 1.5 to 3 years, this improvement in Basis-32 symptom scores was greater than for those who did not engage in co-located treatment (p<0.01). However, there was no change in substance use as measured by the Addiction Severity Index | Over three years of follow-up, there were significant improvements in mental health functioning as assessed by the Kennedy Axis-5 scales, the SF-12 Mental Health Scale, and the Basis-32 Psychosis, Depression and Anxiety scale. Gains were apparent at the 6-month follow-up and were sustained throughout the 3-year follow-up period (all p<0.05). A significant decrease in behaviours that are dangerous to self and others such as angry outbursts, violent acts, suicide attempts, and self-harm as measured by the Kennedy Axis V scale was found for those individuals who remained in the program for 30 months | Not reported | For the entire cohort over three years of follow-up, there was a total decrease of USD$127,350 in the cost of alcohol and drug treatment services, equated to a mean decrease of USD$1,010 per patient. There were overall cost reductions in the following acute and subacute levels of mental health care: emergency services (USD$7,221 reduction), residential services (USD$9,773 reduction), and inpatient psychiatric hospitalisations (USD$46,787 reduction). There was a USD$301,893 increase in physical health-related cost; a mean increase of USD$2,395 per patient. There was also an increase of USD$177,888 in the cost of mental health treatment across the entire cohort - a mean increase of USD$1,412 per patient. This included an increase of USD$198,540 for outpatient mental health services, case management and medication support |
| Kidorf et al. 2013 | Not reported | There were no improvements in both co-located treatment groups in the number of observed opioid positive urine samples (F (3, 345) = 0.8, p=0.50). The proportion of urine samples positive for sedatives increased from the 1- to 3-month follow-ups (0.16 vs. 0.18: F (3, 345) = 2.9, p=0.03) | Both co-located treatment groups showed reductions in SCL-90-R GSI (psychiatric symptom/distress) scores over time (F (3, 317) = 15.5, p<0.001). GSI scores were significantly lower than baseline at all three follow-up time points, and 3-month follow-up GSI scores were significantly lower than at 1-month (p-values not reported) | Not reported | Not reported |
| Lee at al. 2009 | Significantly more at-risk drinkers who received services by the 6-month time point were in the co-located care condition (93% vs 35%; p=0.001). The amount of time between the index assessment and first treatment visit for individuals in the co-located treatment group was reduced (mean = 33 days, SD = 29 days) compared to those in the referral condition (mean = 72 days, SD = 46 days; p=0.03). The mean number of binge drinking episodes in the co-located treatment group decreased significantly from baseline to 6-month follow-ups compared to the standard care group (21.7 reduction and 2.6 increase, respectively; p=0.033) | The co-located care group had significant reductions in drinks per week between baseline and 6 months (mean = 16.8 fewer drinks/week) compared to the standard care group (mean = 0.6 fewer drinks/week; p=0.022). There was no significant difference in alcohol related problems as measured by the Short Michigan Alcohol Screening Test-Geriatric Version (p=0.132) | Not reported | Not reported | Not reported |
| Logan et al. 2019 | Of 61 individuals initiated on buprenorphine, 56 (92%) and 39 (64%) remained in care for 1 and at least 3 months, respectively | Not reported | After adjusting for age, race, and sex, there was a decrease in Patient Health Questionnaire-9 depression symptoms (n = 59, beta = -0.04, 95% CI 0.06, 0.02, p<0.001) and Generalised Anxiety Disorder-7 scale anxiety symptoms (n = 56, beta = -0.04, 95% CI 0.06, 0.02, p<0.0001) across the study period | Not reported | Not reported |
| Mangrum et al. 2006 | Not reported | Not reported | Not reported | The co-located group demonstrated greater reduction in the proportion of participants that had at least one psychiatric hospitalisation during the 12 months pre-baseline and post-baseline, falling from 12-3%, whereas the standard care group demonstrated an increase from 7-11% (p=0.0001). Analyses of the total number of psychiatric hospitalisation days also indicated a significant treatment group by time interaction, with the co-located group displaying a reduction in average total days from 6-2 and the standard care group an increase from an average of 3-6 days (p=0.04). A non-significant trend (p=0.06) was found suggesting a decrease in the total number of psychiatric hospital admissions for the co-located group from an average of 0.17-0.08 compared to a concurrent increase for the standard care group from 0.09-0.16 | Not reported |
| McFall et al. 2006 | Not reported | 41% of participants (n = 44) who received co-located smoking cessation therapy in PTSD clinics achieved abstinence at one or more follow-up intervals. The number of cigarettes smoked per day in the prior month was significantly lower at months 2, 4, 6, and 9 when compared to pre-treatment levels (all p<0.001). Expired carbon monoxide readings taken at all follow-up assessment intervals were significantly lower than baseline (p=0.005); with reductions of 25% at 2 months, 15% at 4 months, 16% at 6 months, and 9% at 9 months. Participants attending six sessions were over three times more likely to achieve repeated seven-day point prevalence abstinence than those attending three or fewer sessions (OR 3.59, 95% CI=1.49, 8.62, p<0.01) | Not reported | Not reported | Not reported |
| Morse et al. 2006 | The non-co-located ACTO condition had significantly more average monthly contacts (means ranging from 6.6 to 7.0 over follow-up) with their clients than both the co-located IACT condition (means ranging from 3.5-4.6 over follow-up) and the control condition (means ranging from 1.3-1.8 over follow-up). The co-located IACT condition had significantly more contact with their clients than the control condition (p<0.001). Participants in the ACTO and IACT conditions had more substance use disorder treatment contacts and were significantly more satisfied with their treatment, respectively, than those in the control condition (p<0.001 and p=0.03) | Not reported | Not reported | Not reported | The IACT and control conditions resulted in lower outpatient costs than ACTO (p<0.001). There was no significant difference in outpatient treatment costs and total costs between IACT and control conditions. Inpatient costs across the three treatment conditions were not significantly different (p=0.90) |
| Morse et al. 2008 | Participants in the co-located NIACT condition had significantly more phone and in-person contacts, respectively, with their treatment team than participants in Integrated ACT, ACTO and standard care conditions (both p<0.001). Participants in the co-located NIACT condition also had more substance use disorder treatment sessions than participants in the other conditions (all p<0.01) | Compared to the ACTO and Integrated Assertive Community Treatment, participants in the co-located NIACT condition had reduced frequency (days) of drug use (both p<0.05) | There was no effect of treatment condition on psychiatric symptoms measured by the BPRS (p=0.19) | Not reported | Not reported |
| Neufeld et al. 2010 | 43% of the sample (n = 35) met criteria for program completion defined as full adherence to scheduled treatment and 2 or more consecutive weeks of urine samples without the detection of drugs. Patients receiving co-located care attended an average of 13 (SD = 8.3, range = 1–41) individual counselling sessions and a mean of 40 (SD = 40.5, range = 0–181) group-based counselling sessions | Not reported | 44% (n = 36/81) of participants had an evaluation provided by a psychiatrist. 83% (n = 30) of these patients with a scheduled evaluation completed it. For those receiving a psychiatric evaluation only with no additional pain evaluation (n = 20), almost all (n = 18) received a diagnosis of a current mental illness. 12 (67%) of these patients were initiated on a psychiatric medication regimen | Not reported | Not reported |
| Rasch et al. 2013 | Individuals receiving co-located dual diagnosis treatment engaged with the service for a mean length of 78 days and 52% of the participants completed the 8-week treatment program | At follow-up, there were numerical decreases in the number of days of substance use in the prior 30 days for all substances. There were statistically significant decreases in the number of days of use of cannabis (p<0.0001), methamphetamine (p<0.0001), and cocaine (p<0.0001) | At follow-up, there were significant reductions compared to baseline in the mean number of days of experiencing the following mental health symptoms: serious depression, serious anxiety, hallucinations, and taking prescribed psychiatric medications (all p<0.05). Furthermore, there were significant decreases in psychological symptoms as measured by the Brief Symptom Inventory. These symptoms included somatisation, obsessive-compulsive, interpersonal sensitivity, depression, anxiety, phobic anxiety, paranoid ideation, psychoticism and hostility (all p<0.05). There was no reduction in the mean number of suicide attempts at follow-up (p>0.05) compared to baseline (prior 30 days). There was no difference in HIV risk behaviours, as measured by unsafe sexual practices, between baseline and follow-up (p=0.058). Quality of life, measured using the California Quality of Life Survey, improved significantly from baseline to follow-up (p<0.05) | Not reported | Not reported |
| Walter et al. 2022 | Not reported | There was a significant decrease in the proportion of participants who reported at least one day of illicit drug use in the prior 30 days between baseline and the 12-month follow-up (p<0.05). However, after adjusting for age, gender, ethnicity, race, education level, and employment status, there was no difference in illicit drug use over the study period (p>0.05) | Depressive symptoms, as measured using the Patient Health Questionnaire-9 (cut-off score ≥ 10), significantly decreased at 6-month (AOR 0.5; 95% CI 0.3-0.8) and at 12-month follow-ups (change in AOR 0.4; 95% CI 0.2-0.7) when compared to baseline. Anxiety symptoms, Generalised Anxiety Disorder-7 scale (cut-off score ≥ 10) also significantly decreased at 6-month (AOR 0.5; 95% CI 0.3-0.9) and at 12-month follow-ups (AOR 0.5; 95% CI 0.3-0.8) when compared to baseline | At 12-month follow-up, participants were approximately 60% less likely to have visited an emergency department in the past 30 days (AOR 0.4, 95% CI 0.2–0.8) compared to baseline | Not reported |
| Xie et al. 2005 | Not reported | Over three years of follow-up, there were significant improvements in scores on the Alcohol Use Scale (p<0.01), Addiction Severity Index alcohol composite (p<0.01), Substance Abuse Treatment Scale (p<0.01), Drug Use Scale (p<0.05) and Addiction Severity Index drug composite (p<0.05). Measured using the Timeline follow-back method, the mean number of days of alcohol use over the prior six months decreased from 60 at baseline to 36 at the 3-year follow-up (p<0.01). The proportion of participants that achieved full remission over the past 6 months increased from 1% at baseline to 40% at the 3-year follow-up (p<0.01) | Psychiatric symptoms, assessed with the Expanded BPRS improved significantly from baseline (47.7) to the 3-year follow-up (42.6; p<0.01). Participants reported greater general life satisfaction on the Quality-of-Life Interview Satisfaction scale over follow-up time compared to baseline (p<0.01) | Over the follow-up period, the mean hours of outpatient case management contacts and medication visits increased significantly from baseline (both p<0.01). There was no significant difference in the mean number of hospitalisation days over the study period (p>0.05) | Not reported |

ACT, Assertive Community Treatment; ACTO, Assertive Community Treatment only; ANOVA, analysis of variance; AOR, adjusted odds ratio; BPRS, Brief Psychiatric Rating Scale; CI, confidence interval; DSM-III-R, DSM-III-R, Diagnostic and Statistical Manual of Mental Disorders, Third Edition, Revised; ED,emergency department; GSI, Global Severity Index; IACT, Integrated Assertive Community Treatment; NIACT, New Integrated Assertive Community Treatment; OR, odds ratio; PTSD, post-traumatic stress disorder; SCL-90-R GSI, Symptom Checklist – Global Severity Index; SE, standard error; SSRI, selective serotonin reuptake inhibitors.

## **Table S9. Assessment of risk of bias for individual studies reporting health outcomes and service use patterns associated with co-located outpatient mental health and alcohol and other drug specialist treatment**

| **Unique safeguards** | **Bartels et al. 2004** | **Bhalla et al. 2020** | **Bond et al. 1991** | **Bouchery et al. 2018** | **Brooner et al. 2013** | **Clark et al. 1998** | **Clausen et al. 2021** | **Cooper et al. 2010** | **Drake et al. 1993** | **Drake et al. 1998** | **Drake et al. 2004** | **Drake et al. 2016** | **Fletcher et al. 2008** | **Frisman et al. 2009** | **Granholm et al. 2003** | **Holdcraft et al. 2002** | **Judd et al. 2003** | **Kidorf et al. 2013** | **Lee et al. 2009** | **Logan et al. 2019** | **Mangrum et al. 2006** | **McFall et al. 2006** | **Morse et al. 2006** | **Morse et al. 2008** | **Neufeld et al. 2010** | **Rasch et al. 2013** | **Walter et al. 2019** | **Xie et al. 2005** |
| --- | --- | --- | --- | --- | --- | --- | --- | --- | --- | --- | --- | --- | --- | --- | --- | --- | --- | --- | --- | --- | --- | --- | --- | --- | --- | --- | --- | --- |
| 1. Further selection after study start unrelated to intervention or tendency for development of the outcome or did not happen | 1 | 1 | 0 | 1 | 1 | 1 | 1 | 1 | 0 | 1 | 1 | 1 | 1 | 1 | 0 | 0 | 1 | 1 | 1 | 1 | 1 | 1 | 1 | 0 | 0 | 1 | 0 | 1 |
| 2. Groups were created a priori from the same population and timeframe (recruitment) | 1 | 0 | 0 | 0 | 1 | 0 | 1 | 1 | 1 | 1 | 1 | 1 | 1 | 0 | 0 | 1 | 1 | 1 | 1 | 0 | 1 | 1 | 1 | 0 | 1 | 1 | 1 | 0 |
| 3. Inclusions/exclusion criteria objectively defined and applied equally to all groups prior to study recruitment | 1 | 0 | 1 | 1 | 1 | 1 | 1 | 1 | 0 | 1 | 1 | 1 | 1 | 0 | 0 | 1 | 1 | 1 | 1 | 1 | 1 | 1 | 1 | 1 | 1 | 1 | 1 | 1 |
| 4. Any attrition (or exclusions after entry) were described and not markedly different between groups or did not happen | 1 | 0 | 0 | 1 | 1 | 0 | 1 | 0 | 1 | 0 | 1 | 0 | 0 | 0 | 1 | 0 | 1 | 1 | 0 | 0 | 0 | 1 | 1 | 0 | 1 | 0 | 0 | 1 |
| 5. Loss to follow-up or missing data <15% or no missing data | 1 | 0 | 0 | 1 | 1 | 1 | 0 | 0 | 0 | 1 | 0 | 0 | 0 | 0 | 1 | 0 | 0 | 0 | 0 | 0 | 0 | 0 | 0 | 0 | 0 | 0 | 0 | 1 |
| 6. Assessment methods were the same for all groups | 1 | 1 | 1 | 1 | 1 | 1 | 1 | 1 | 1 | 1 | 1 | 1 | 1 | 1 | 1 | 1 | 1 | 1 | 1 | 1 | 1 | 1 | 1 | 1 | 1 | 1 | 1 | 1 |
| 7. Intervention/exposure follow-up captures all intervention/exposure time | 0 | 0 | 1 | 1 | 1 | 1 | 1 | 0 | 1 | 1 | 1 | 1 | 1 | 1 | 1 | 1 | 1 | 1 | 1 | 1 | 1 | 1 | 1 | 1 | 1 | 1 | 1 | 1 |
| 8. Outcome/case definition was objectively defined or a reference standard used or a hard outcome | 1 | 1 | 1 | 1 | 1 | 1 | 1 | 1 | 1 | 1 | 1 | 1 | 1 | 1 | 1 | 1 | 1 | 1 | 1 | 1 | 1 | 1 | 1 | 1 | 1 | 1 | 1 | 1 |
| 9. Analyst was blinded | 0 | 0 | 0 | 0 | 0 | 0 | 0 | 0 | 0 | 0 | 1 | 0 | 0 | 0 | 0 | 0 | 0 | 0 | 0 | 0 | 0 | 0 | 0 | 0 | 0 | 0 | 0 | 0 |
| 10. Outcome assessor(s) were blinded | 0 | 0 | 0 | 0 | 0 | 0 | 0 | 0 | 0 | 0 | 0 | 0 | 0 | 1 | 0 | 0 | 0 | 0 | 0 | 0 | 0 | 0 | 0 | 0 | 0 | 0 | 0 | 1 |
| 11. Duration and intensity of intervention/exposure was sufficient to expect an effect | 1 | 1 | 1 | 1 | 1 | 1 | 1 | 1 | 1 | 1 | 1 | 1 | 1 | 1 | 1 | 1 | 1 | 1 | 1 | 1 | 1 | 1 | 1 | 1 | 1 | 1 | 1 | 1 |
| 12. Follow-up period was sufficient to expect intervention effects | 1 | 1 | 1 | 1 | 1 | 1 | 1 | 1 | 1 | 1 | 1 | 1 | 1 | 1 | 1 | 1 | 1 | 1 | 1 | 1 | 1 | 1 | 1 | 1 | 1 | 1 | 1 | 1 |
| 13. Outcome assessed from participants not proxy (e.g. records) | 0 | 0 | 0 | 0 | 1 | 1 | 1 | 1 | 0 | 1 | 1 | 1 | 1 | 1 | 1 | 0 | 1 | 1 | 0 | 0 | 0 | 0 | 1 | 1 | 0 | 1 | 1 | 1 |
| 14. Participants were blinded | 0 | 0 | 0 | 0 | 0 | 0 | 0 | 0 | 0 | 0 | 0 | 0 | 0 | 0 | 0 | 0 | 0 | 0 | 0 | 0 | 0 | 0 | 0 | 0 | 0 | 0 | 0 | 0 |
| 15. Primary outcome measures were specified a priori | 1 | 0 | 1 | 0 | 1 | 1 | 1 | 1 | 1 | 1 | 1 | 1 | 1 | 1 | 0 | 1 | 1 | 1 | 0 | 0 | 1 | 1 | 1 | 0 | 1 | 1 | 1 | 1 |
| 16. Carers were blinded | 0 | 0 | 0 | 0 | 0 | 0 | 0 | 0 | 0 | 0 | 0 | 0 | 0 | 0 | 0 | 0 | 0 | 0 | 0 | 0 | 0 | 0 | 0 | 0 | 0 | 0 | 0 | 0 |
| 17. Care was delivered equally to both groups | 0 | 0 | 0 | 0 | 0 | 0 | 1 | 0 | 1 | 0 | 0 | 0 | 0 | 0 | 0 | 1 | 1 | 1 | 0 | 1 | 0 | 0 | 0 | 0 | 0 | 0 | 0 | 1 |
| 18. Cointerventions that could impact the outcome were comparable between groups or avoided | 0 | 0 | 0 | 0 | 0 | 0 | 0 | 0 | 0 | 0 | 0 | 0 | 1 | 1 | 0 | 0 | 0 | 0 | 0 | 0 | 1 | 0 | 0 | 0 | 0 | 0 | 0 | 0 |
| 19. Control exposure measured reliably, or placebo used and there were no contrived comparisons | 1 | 0 | 1 | 1 | 1 | 1 | 1 | 0 | 0 | 1 | 0 | 1 | 1 | 1 | 1 | 1 | 0 | 1 | 1 | 0 | 0 | 1 | 1 | 1 | 0 | 1 | 1 | 0 |
| 20. Active exposure measured reliably, or intervention controlled by investigator | 1 | 1 | 1 | 1 | 1 | 1 | 1 | 1 | 1 | 1 | 0 | 1 | 1 | 1 | 1 | 1 | 1 | 1 | 1 | 0 | 0 | 1 | 1 | 1 | 1 | 1 | 1 | 1 |
| 21. Exposures measured in the same way for all participants (e.g., dose/type/potency) | 1 | 0 | 1 | 1 | 1 | 1 | 1 | 1 | 0 | 1 | 1 | 1 | 1 | 1 | 1 | 1 | 1 | 1 | 1 | 1 | 1 | 1 | 1 | 1 | 1 | 1 | 1 | 1 |
| 22. Outcome criteria consistently applied | 1 | 1 | 1 | 1 | 1 | 1 | 1 | 1 | 0 | 1 | 1 | 1 | 1 | 1 | 1 | 1 | 1 | 1 | 1 | 1 | 1 | 1 | 1 | 1 | 1 | 1 | 1 | 1 |
| 23. The time period between exposure and outcome is similar across patients and between groups or the analyses adjust for different lengths of follow-up of patients | 1 | 0 | 1 | 0 | 1 | 1 | 1 | 1 | 1 | 1 | 1 | 1 | 1 | 1 | 1 | 1 | 1 | 1 | 1 | 1 | 1 | 1 | 1 | 1 | 1 | 1 | 1 | 1 |
| 24. Treatment deviations or noncompliance/nonadherence were <20%/treatment delivered as allocated in experimental studies or observational study reported clearly that there were minimal treatment deviations | 1 | 0 | 1 | 0 | 1 | 1 | 0 | 0 | 0 | 1 | 0 | 0 | 0 | 0 | 0 | 0 | 0 | 1 | 1 | 1 | 0 | 1 | 0 | 0 | 0 | 0 | 0 | 1 |
| 25. Analytic strategies in place (e.g., covariate adjustment, propensity score or instrumental variable) to avoid confounding (including confounding by indication) or participants randomised and no exclusions after randomisation | 1 | 1 | 0 | 0 | 1 | 1 | 1 | 0 | 0 | 1 | 0 | 1 | 1 | 0 | 0 | 0 | 0 | 1 | 0 | 0 | 0 | 0 | 0 | 0 | 0 | 0 | 1 | 0 |
| 26. Analytic strategy for time-varying confounding was addressed or there was no need to address time-varying confounding | 1 | 1 | 0 | 0 | 0 | 0 | 0 | 1 | 0 | 1 | 1 | 0 | 1 | 1 | 1 | 0 | 0 | 0 | 0 | 0 | 0 | 0 | 0 | 0 | 0 | 0 | 0 | 0 |
| 27. ITT/LATE/CACE/per-protocol/on-treatment analyses used for treatment deviations (experimental study) or not required or protocol clearly defined in observational study or analysis accounted for missing data | 1 | 0 | 0 | 1 | 1 | 0 | 1 | 0 | 0 | 1 | 0 | 0 | 0 | 0 | 0 | 0 | 0 | 0 | 0 | 1 | 0 | 1 | 1 | 0 | 0 | 0 | 0 | 0 |
| 28. Key baseline characteristics/prognostic indicators for the study were comparable across groups | 1 | 0 | 0 | 1 | 1 | 1 | 1 | 1 | 1 | 0 | 1 | 1 | 1 | 1 | 1 | 1 | 1 | 1 | 0 | 1 | 0 | 1 | 0 | 0 | 0 | 1 | 1 | 1 |
| 29. Allocation procedure was adequate and concealed | 0 | 0 | 0 | 0 | 0 | 0 | 0 | 0 | 0 | 1 | 0 | 0 | 0 | 0 | 0 | 0 | 0 | 1 | 1 | 0 | 0 | 0 | 0 | 0 | 0 | 0 | 0 | 0 |
| 30. Conflict of interests were declared and absent | 0 | 1 | 0 | 1 | 1 | 0 | 1 | 0 | 0 | 0 | 0 | 1 | 0 | 0 | 0 | 0 | 0 | 1 | 1 | 0 | 0 | 0 | 0 | 0 | 0 | 1 | 1 | 0 |
| 31. Participants were randomly allocated to groups with adequate randomisation process (if participants own control then treatment order randomised) | 1 | 0 | 0 | 0 | 1 | 0 | 0 | 0 | 0 | 1 | 0 | 0 | 1 | 0 | 0 | 0 | 0 | 1 | 1 | 0 | 0 | 0 | 0 | 0 | 0 | 0 | 0 | 1 |
| 32. Analytic method justified by study design (e.g., RR not used in a case-control design or cross-over designs properly handled etc.) | 1 | 1 | 1 | 1 | 1 | 1 | 1 | 1 | 0 | 1 | 1 | 1 | 1 | 1 | 1 | 0 | 1 | 1 | 1 | 1 | 1 | 1 | 1 | 1 | 1 | 0 | 1 | 1 |
| 33. Computation errors or contradictions were absent | 1 | 1 | 1 | 1 | 1 | 1 | 1 | 1 | 1 | 1 | 0 | 1 | 1 | 1 | 1 | 1 | 1 | 1 | 1 | 1 | 1 | 1 | 1 | 1 | 1 | 1 | 1 | 1 |
| 34. There was not data dredging (including decision to validate results not dependent on findings of the study) | 1 | 1 | 1 | 1 | 1 | 1 | 1 | 1 | 1 | 1 | 1 | 0 | 0 | 1 | 1 | 1 | 1 | 1 | 1 | 0 | 1 | 1 | 1 | 1 | 1 | 1 | 1 | 1 |
| 35. All participants were selected prior to outcome development and evaluated prospectively | 1 | 0 | 1 | 1 | 1 | 0 | 1 | 1 | 0 | 1 | 1 | 1 | 1 | 0 | 0 | 1 | 1 | 1 | 1 | 0 | 1 | 1 | 1 | 1 | 0 | 0 | 0 | 0 |
| 36. Carry-over or refractory effects were avoided or considered in the design of the study or were not relevant | 1 | 0 | 1 | 1 | 1 | 1 | 1 | 1 | 1 | 1 | 1 | 1 | 1 | 1 | 1 | 1 | 1 | 1 | 1 | 1 | 1 | 1 | 1 | 1 | 1 | 1 | 1 | 1 |
| 37. Response rate to participant request was greater than 80% or no difference between responders and non-responders | 0 | 1 | 0 | 1 | 1 | 0 | 0 | 0 | 0 | 1 | 0 | 0 | 0 | 0 | 0 | 0 | 0 | 0 | 0 | 0 | 0 | 0 | 0 | 0 | 0 | 0 | 0 | 0 |
| 38. Some form of random sampling used or sampling appropriate | 1 | 1 | 1 | 1 | 1 | 0 | 0 | 0 | 0 | 1 | 0 | 0 | 1 | 0 | 0 | 0 | 0 | 0 | 0 | 1 | 1 | 0 | 0 | 1 | 1 | 0 | 1 | 1 |
| 39. Study sampling frame population is similar to the target population | 1 | 1 | 1 | 1 | 1 | 0 | 0 | 1 | 0 | 1 | 0 | 0 | 0 | 0 | 1 | 0 | 1 | 1 | 1 | 1 | 1 | 1 | 1 | 1 | 1 | 1 | 1 | 1 |
| 40. The study target population is indeed the group of people or entities to which the results of the study will be generalised | 1 | 1 | 1 | 0 | 1 | 1 | 1 | 0 | 1 | 1 | 1 | 1 | 0 | 0 | 1 | 0 | 1 | 1 | 1 | 1 | 1 | 1 | 1 | 1 | 1 | 1 | 1 | 1 |
| **Total** | **29** | **17** | **21** | **24** | **32** | **23** | **27** | **21** | **16** | **31** | **22** | **23** | **25** | **21** | **21** | **19** | **24** | **30** | **24** | **20** | **21** | **25** | **24** | **20** | **20** | **22** | **24** | **27** |

CACE, Complier Average Causal Effect; ITT, Intention To treat; LATE, Local Average Treatment Effect; RR: Relative Risk

**References:**

1. Bartels SJ, Coakley EH, Zubritsky C, Ware JH, Miles KM, Areán PA, et al. Improving access to geriatric mental health services: a randomized trial comparing treatment engagement with integrated versus enhanced referral care for depression, anxiety, and at-risk alcohol use. Am J Psychiatry. 2004;161:1455-62.

2. Brooner RK, Kidorf MS, King VL, Peirce J, Neufeld K, Stoller K, et al. Managing psychiatric comorbidity within versus outside of methadone treatment settings: a randomized and controlled evaluation. Addiction. 2013;108:1942-51.

3. Lee HS, Mericle AA, Ayalon L, Areán PA. Harm reduction among at‐risk elderly drinkers: a site‐specific analysis from the multi‐site Primary Care Research in Substance Abuse and Mental Health for Elderly (PRISM‐E) study. Int J Geriatr Psychiatry. 2009;24:54-60.

4. Morse GA, Calsyn RJ, Klinkenberg WD, Helminiak TW, Wolff N, Drake RE, et al. Treating homeless clients with severe mental illness and substance use disorders: costs and outcomes. Community Ment Health J. 2006;42:377-404.

5. Judd PH, Thomas N, Schwartz T, Outcalt A, Hough R. A dual diagnosis demonstration project: Treatment outcomes and cost analysis. J Psychoactive Drugs. 2003;35(Suppl 1):181-92.

6. Walter AW, Morocho C, Chassler D, Sousa J, De Jesús D, Longworth-Reed L, et al. Evaluating culturally and linguistically integrated care for Latinx adults with mental and substance use disorders. Ethn Health. 2022;27:407-19.

7. Xie H, McHugo GJ, Helmstetter BS, Drake RE. Three-year recovery outcomes for long-term patients with co-occurring schizophrenic and substance use disorders. Schizophr Res. 2005;75:337-48.

8. Drake RE, McHugo GJ, Clark RE, Teague GB, Xie H, Miles K, et al. Assertive community treatment for patients with co-occurring severe mental illness and substance use disorder: A clinical trial. Am J Orthopsychiatry. 1998;68:201-15.

9. Fletcher TD, Cunningham JL, Calsyn RJ, Morse GA, Klinkenberg WD. Evaluation of treatment programs for dual disorder individuals: modeling longitudinal and mediation effects. Adm Policy Ment Health. 2008;35:319-36.

10. Kidorf M, Brooner RK, Gandotra N, Antoine D, King VL, Peirce J, et al. Reinforcing integrated psychiatric service attendance in an opioid-agonist program: a randomized and controlled trial. Drug Alcohol Depend. 2013;133:30-6.

11. McFall M, Atkins DC, Yoshimoto D, Thompson CE, Kanter E, Malte CA, et al. Integrating tobacco cessation treatment into mental health care for patients with posttraumatic stress disorder. Am J Addict. 2006;15:336-44.

12. Clausen H, Odden S, Ruud T, Benth JS, Heiervang KS, Stuen HK, et al. Improved rehabilitation outcomes for persons with and without problematic substance use after 2 years with assertive community treatment-A prospective study of patients with severe mental illness in 12 Norwegian ACT teams. Front Psychiatry. 2020;11:607071.

13. Bond GR, McDonel EC, Miller LD, Pensec M. Assertive community treatment and reference groups: An evaluation of their effectiveness for young adults with serious mental illness and substance abuse problems. Psychosoc Rehabil J. 1991;15:31.

14. Holdcraft LC, Comtois KA. Description of and preliminary data from a women's dual diagnosis community mental health program. Can J Commun Ment Health. 2009;21:91-109.

15. Bouchery EE, Siegwarth AW, Natzke B, Lyons J, Miller R, Ireys HT, et al. Implementing a whole health model in a community mental health center: Impact on service utilization and expenditures. Psychiatr Serv. 2018;69:1075-80.

16. Young JT, Heffernan E, Borschmann R, Ogloff JRP, Spittal MJ, Kouyoumdjian FG, et al. Dual diagnosis of mental illness and substance use disorder and injury in adults recently released from prison: a prospective cohort study. Lancet Public Health. 2018;3:e237-48.

17. National Centre for Classification in Health. International statistical classification of diseases and related health problems, Tenth Revision, Australian Modification (ICD-10-AM). National Centre for Classification in Health, Faculty of Health Sciences, University of Sydney; 2004.
